# A new class of inhibitors of the AraC family virulence regulator *Vibrio cholerae* ToxT

**DOI:** 10.1038/srep45011

**Published:** 2017-03-23

**Authors:** Anne K. Woodbrey, Evans O. Onyango, Maria Pellegrini, Gabriela Kovacikova, Ronald K. Taylor, Gordon W. Gribble, F. Jon Kull

**Affiliations:** 1 Department of Chemistry, Dartmouth College, Hanover, NH, 03755, USA; 2 Department of Microbiology and Immunology, Geisel School of Medicine at Dartmouth, Hanover, NH, 03755, USA

## Abstract

*Vibrio cholerae* is responsible for the diarrheal disease cholera that infects millions of people worldwide. While vaccines protecting against cholera exist, and oral rehydration therapy is an effective treatment method, the disease will remain a global health threat until long-term solutions such as improved sanitation and access to clean water become widely available. Because of this, there is a pressing need for potent therapeutics that can either mitigate cholera symptoms, or act prophylactically to prevent the virulent effects of a cholera infection. Here we report the design, synthesis, and characterization of a set of compounds that bind and inhibit ToxT, the transcription factor that directly regulates the two primary *V. cholerae* virulence factors. Using the folded structure of the monounsaturated fatty acid observed in the X-ray structure of ToxT as a template, we designed ten novel compounds that inhibit the virulence cascade to a greater degree than any known inhibitor. Our findings provide a structural and functional basis for the development of viable antivirulence therapeutics that combat cholera and, potentially, other forms of bacterial pathogenic disease.

Enteric diarrheal disease continues to be a global health concern and is especially deadly to children in third world countries^1^,^2^. Ironically, the most effective method of treatment – water – is also responsible for transmission: while oral rehydration therapy is highly effective, contaminated water harbors the diarrheal bacteria and spreads infection^1^. Yet, despite the fact that access to clean water is a simple solution to diarrheal disease, parts of the world stricken by natural and civil disasters often see an upsurge in cholera cases, and outbreaks are frequent and ongoing. The emergence of drug resistant bacterial strains and the inevitability of natural disasters add to the complexity of the problem. There is, therefore, immediate need for effective therapeutics against enteric bacterial infections that do not lead to increased resistance and are simple to deploy, prompting the current study.

Gastrointestinal infection caused by the ingestion of contaminated food or water is the primary cause of enteric diarrheal disease. While many enteric bacteria are acid labile, pathogens that survive conditions in the stomach^3^,^4^,^5^,^6^ go on to produce virulence factors through a chain of transcriptional events initiated by environmental stimuli. Production of these virulence factors, including toxins and adhesion factors, ultimately results in diarrhea and other hallmarks of pathogenicity^7^. In the case of *Vibrio cholerae*, colonization depends on the toxin-coregulated pilus (TCP), which aggregates *V. cholerae* bacteria and induces microcolony formation^8^. The bacteria then express cholera toxin (CT), an exotoxin internalized into the host epithelial cells that disrupts ion transport and results in secretion of water into the lumen of the intestine^9^,^10^. Regulation of TCP and CT, the primary virulence factors of *V. cholerae*, depends primarily on the transcriptional activator ToxT^11^, a member of the large AraC/XylS family^12^,^13^. ToxT directly activates TCP and CT expression and thus is commonly referred to as the master regulator of *V. cholerae* virulence^14^,^15^.

Investigation of the environmental factors influencing *V. cholerae* virulence, such as amino acids and pH, dates back to the 1980s^16^. It was later suggested that bile had an effect on the ToxR regulon^17^, and, more specifically, on ToxT-dependent expression of CT and TCP^18^. Since then, our understanding of such effects has expanded, as has the goal to identify and exploit specific mechanisms involving ToxT regulation. The inhibitory effects of bile on *V. cholerae* virulence were eventually attributed to its unsaturated fatty acid (UFA) components^19^. While these findings documented inhibition of ToxT-activated gene expression by UFAs, a direct link between ToxT and fatty acids was not revealed until the X-ray structure was solved^20^. The presence of *cis*-palmitoleate, a sixteen carbon monounsaturated fatty acid, in a pocket within the regulatory domain of ToxT suggested a fatty acid-mediated mode of inhibition and that oleic acid, a major component of bile^19^, could be the natural ToxT inhibitor^20^. Subsequent studies have shown that additional UFAs, including linoleic and conjugated linoleic acid, also inhibit ToxT^19^,^20^,^21^,^22^. The small molecule virstatin has also been shown to inhibit ToxT activity by preventing dimerization^23^,^24^. However, the concentrations required to reduce colonization in the infant mouse model were relatively high, and it has been shown that virstatin is not effective against some non-O1/non-O139 *V. cholerae* strains^23^,^25^.

In order to develop a more potent ToxT inhibitor, we have taken a structure-based approach to design a set of compounds that inhibit the *V. cholerae* virulence cascade. In this study, we used the folded conformation of the UFA ligand as inspiration to design a general bicyclic compound that will serve as the template for increasingly effective ToxT inhibitors. Our goal is to synthesize and characterize chemical derivatives of this model compound in order to determine the critical chemical characteristics responsible for inhibition. We present evidence that our most potent small-molecule inhibitors inhibit expression of essential colonization genes at 50 nM concentrations. These compounds represent a set of potential drug therapeutics that we show to be the most effective inhibitors of ToxT-regulated virulence gene expression described to date.

## Results

### Rational design of small-molecule inhibitors

**Figure 1:**
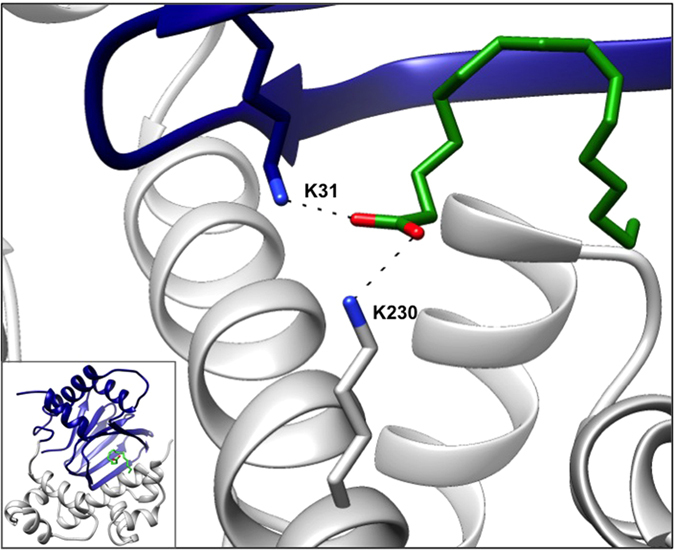
The fatty acid-binding region of ToxT. The carboxylate head of *cis*-palmitoleate interacts with Lys31 of the N-terminal regulatory domain and Lys230 of the C-terminal DNA-binding domain. The full structure of ToxT is shown in the inset (PDB 3GBG). Blue, N-terminal domain; grey, C-terminal domain; green, fatty acid.

The X-ray structure of ToxT revealed a 16-carbon monounsaturated fatty acid *cis*-palmitoleate bound in a pocket within the N-terminal domain^20^,^26^, the homologous location as the arabinose-binding site within AraC^27^, a related protein for which the family is named. The fatty acid bridges the interface between the N-terminal dimerization domain and C-terminal DNA-binding domain of ToxT, suggesting a potential mechanism for fatty acid-mediated inhibition (Fig. 1). The long aliphatic chain abuts hydrophobic residues within the N-terminal domain and helices 9 and 10 of the C-terminal domain, occupying the bulk of the binding pocket. The anionic carboxylate of *cis*-palmitoleate forms salt bridges with C-terminal and N-terminal lysine residues, presumably locking ToxT in a “closed” conformation in which it is unable to dimerize and/or bind DNA^20^. It has also been suggested that ToxT is protected from proteolysis while in this inactive form^28^. According to current models^20^, upon the release of the fatty acid, repulsive forces between the neighboring lysine residues drive the N- and C-terminal domains apart. This results in an “open” state, freeing the C-terminal domain to bind DNA and/or allowing the N-terminal domain to dimerize^20^. It has been suggested that this rearrangement may involve the flexible N-terminal loop between residues 108–113^26^. Therefore, ToxT is thought to exist in “closed” (inactive) and “open” (active) states^20^.

**Figure 2:**
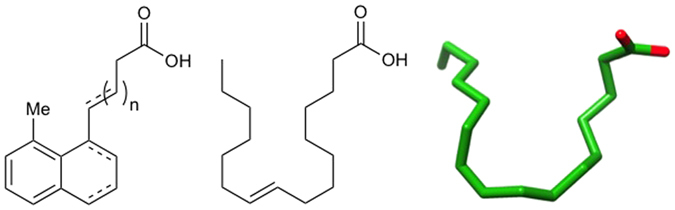
Small-molecule inhibitors resemble folded fatty acids. General structure of the small-molecule inhibitors (left) compared to the 2D representation of *cis*-palmitoleic acid (middle) and the bound conformation of *cis*-palmitoleate from the X-ray crystal structure (right).

This model implies that conformational changes in the ligand-binding pocket and domain interface, resulting from the presence or absence of UFA, influences the DNA-binding domain and/or the dimerization domain. Thus, we hypothesize that the carboxylate head and hydrophobic tail of *cis*-palmitoleate responsible for inter-domain interactions is critical for ToxT inhibition, and maintaining fatty acid-like character is required for an effective *V. cholerae* anti-virulence drug. Using the bound conformation of *cis*-palmitoleate as a template, we have designed a set of small-molecule inhibitors with these general characteristics (Fig. 2). Our design utilizes a fused ring system that should rigidify the synthetic compounds and lead to tighter binding. In contrast to the natural ligand, which must assume a constrained fold in the binding pocket, our “pre-folded” bicyclic compounds have already overcome this entropic penalty. The initial set of compounds includes variations in both the length of the carboxylate chain and degree of saturation of the ring system (Supplementary Fig. S1). We expect further modifications like these may optimize our design and allow the compounds to bind more tightly to the ToxT effector-binding pocket. For example, the affinity of the compounds may be improved by reduction of the tetralin to the corresponding decalin, and/or replacing the methyl group with a larger substituent (i.e. ethyl or *t*-butyl).

### Synthesis of small-molecule inhibitors

**Figure 3:**
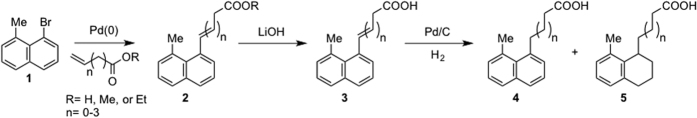
Synthetic scheme. The general synthetic procedure uses Heck coupling, ester hydrolysis, and hydrogenation to give the various small-molecule inhibitors.

The small-molecule inhibitors, whose overall structures resemble compounds **3–5**, were synthesized according to the scheme in Fig. 3. The five- and six-step syntheses of the naphthalene **3**, **4** and tetralin **5** analogs, respectively, utilized the well-known Mizoroki-Heck reaction^29^. The “anchor” for the palladium-catalyzed C-C coupling was 1-bromo-8-methylnaphthalene (**1**), whose synthesis we have previously optimized^30^. Following the attachment of the carboxylic acid chain to the naphthalene ring and hydrolysis of the ester **2** (if necessary), controlled catalytic hydrogenation gave the various final products, with the reaction time corresponding to the level of saturation; hydrogenation of the double bond in the carboxylate chain of **3** occurred after ~30 minutes to yield **4**, while prolonged hydrogenation (overnight) yielded **5**. (See Supplementary Information for detailed synthetic procedures and Supplementary Fig. S1 for the structures of all ten synthesized compounds.)

### Inhibitors prevent virulence factor expression and ToxT-DNA binding

**Figure 4:**
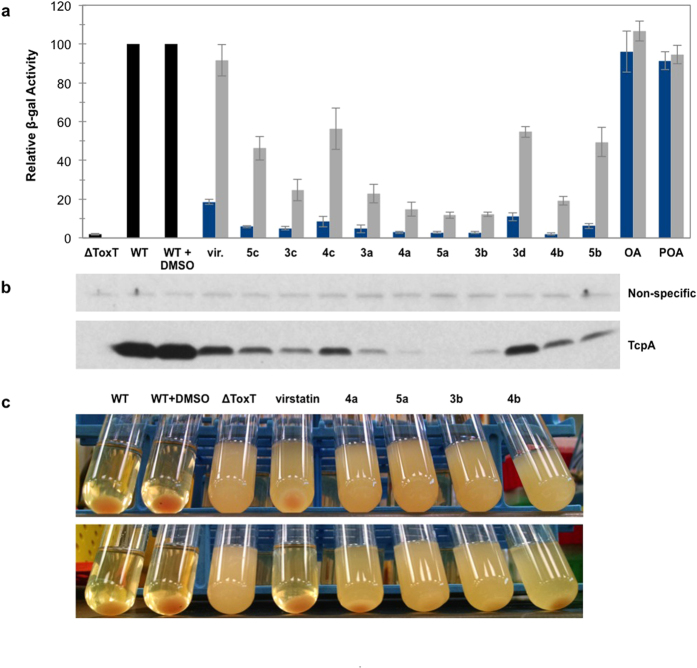
Synthesized compounds inhibit *tcpA* expression and autoagglutination activities. (**a**) Relative β-galactosidase activity of tcpA-lacZ fusion construct in the presence of virstatin (vir.), the ten synthesized compounds, oleic acid (OA), or palmitoleic acid (POA) at concentrations of 5 μM (blue) and 0.5 μM (grey). Relative β-gal activity was calculated as a percentage of the untreated wild-type (WT) strain ± DMSO. ΔToxT is calculated relative to WT. Other values are calculated relative to WT + DMSO. Error bars represent standard deviation where n is 4. The average β-galactosidase activity (in Miller Units) for the WT samples in the absence and presence of DMSO are 15808 ( ± 1120) and 15986 ( ± 495) where n is 5. (**b**) Western blot showing TcpA production in the corresponding lanes. Compounds were added to a final concentration of 5 μM. Fatty acids were not tested. The non-specific band is a loading control. (**c**) Autoagglutination of O395 cultures grown in the presence of 0.5 μM (top) and 0.05 μM (bottom) compounds 4a, 5a, 3b, and 4b.

Initial screening of the synthetic inhibitors in a *V. cholerae* classical biotype transcriptional fusion system (*tcpA-lacZ*) revealed marked inhibition of *tcpA* expression, as measured in a β-galactosidase reporter assay (Fig. 4a). All ten compounds inhibited *tcpA* transcription significantly more than virstatin, a molecule known to inhibit expression of *V. cholerae* virulence factors^23^, whereas *cis*-palmitoleic and oleic acids had essentially no effect at these concentrations (Fig. 4a). At 5 μM, the strongest inhibitors decreased *tcpA-lacZ* transcription levels to almost baseline (that of *ΔToxT tcpA-lacZ*), but did not affect the number of colony forming units (data not shown). Expression of TcpA by Western Blot provided further evidence for the potency of our inhibitors, as the degree of inhibition by each compound was consistent with that determined by the β-galactosidase assay (Fig. 4b). Similarly, compounds **4a**, **5a**, **3b**, and **4b** completely abolished detectable autoagglutination of O395 cultures at 0.5 μM, while virstatin did not (Fig. 4c). When cells are grown under inducing conditions, the production of TCP pili allows for the formation of microcolonies, clusters of bacterial cells that are tethered together and which form a pellet in the bacterial culture^31^,^32^. This process is dependent on production of the major pilin subunit TcpA^31^. Autoagglutination was inhibited by compounds **4a**, **5a**, **3b**, and **4b** even at concentrations as low as 50 nM (Fig. 4c), potentially via inhibition of pili formation. As with the β-gal assay, *cis*-palmitoleic and oleic fatty acids had no effect on *tcpA* transcription or autoagglutination at these concentrations (Supplementary Fig. S2).

**Figure 5:**
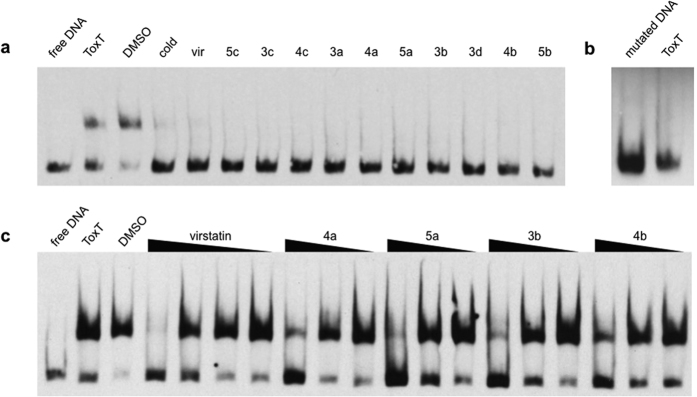
Synthesized compounds inhibit ToxT-DNA binding interactions. (**a**) ToxT EMSA in the presence of virstatin and the compounds. All lanes contain a DIG labeled, 84-bp segment of the *tcpA* promoter. All lanes except lane 1 contain 0.78 μM ToxT. The solvent (DMSO) does not inhibit DNA binding (lane 3). A 100 molar excess of the unlabeled “cold” DNA segment competes for binding (lane 4). The presence of 100 μM virstatin (vir) or 100 μM compounds inhibits DNA binding (lanes 5–15). (**b**) ToxT does not shift the negative control CJ2.6 DNA^35^, a mutated segment of the *tcpA* promoter which ToxT cannot bind. (**c**) ToxT EMSA in the presence of virstatin and four lead compounds. All lanes contain labeled DNA. Virstatin is tested at 100, 10, 1, and 0.1 μM concentrations. Compounds 4a, 5a, 3b and 4b are each tested at 10, 1, and 0.1 μM. The EMSAs shown are representative of three or more independent experiments.

It has been shown that virstatin and UFAs act on ToxT directly by affecting its ability to dimerize and/or bind DNA^20^,^33^. To determine if our newly synthesized compounds acted in a similar manner, electrophoretic mobility shift assays (EMSAs) were performed using purified ToxT protein and a digoxigenin-labeled 84 base-pair segment of the *tcpA* promoter^34^. At 100 μM, all ten compounds and virstatin prevented ToxT from binding DNA (Fig. 5a). As expected, the same concentration of ToxT did not shift a similar mutated probe (Fig. 5b). It is known from prior studies that ToxT is unable to bind this probe due to two point mutations in the *tcpA* promoter^35^. The compounds with greatest inhibition of *tcpA* expression as determined by the β-galactosidase assay were assessed further in order to determine the concentration threshold required for inhibition. Compounds **4a**, **5a**, **3b**, and **4b** inhibited DNA-binding more strongly than virstatin, as seen in the dose-response EMSA (Fig. 5c).

### X-ray crystal structures of ToxT-inhibitor complexes and computational modeling

**Figure 6:**
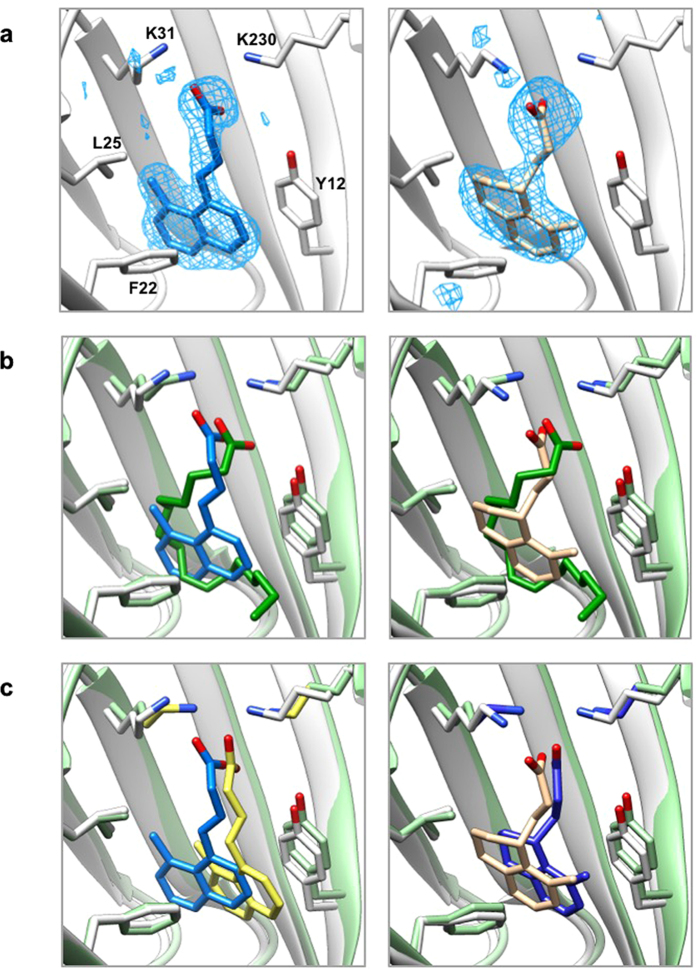
X-ray crystal structures of ToxT-inhibitor complexes reveal compounds bind in the fatty acid-binding pocket. (**a**) Simulated annealing *F*_*o*_ *– F*_*c*_ omit maps of ToxT bound to compounds 3b (left) and 5a (right) contoured at 2.5 σ. (**b**) Overlay of crystal structures of ToxT bound to fatty acid with ToxT bound to 3b (left) and ToxT bound to 5a (right). (**c**) Overlay of crystal structures of ToxT bound to 3b (left) and ToxT bound to 5a (right) with the conformations predicted by AutoDock. Pale green, ToxT bound to fatty acid (dark green, PDB ID 3GBG); grey, ToxT bound to compounds 3b (blue, PDB ID 5SUX) and 5a (tan, PDB ID 5SUW); yellow, bound confirmation of compound 3b as predicted by AutoDock; dark blue, bound confirmation of compound 5a as predicted by AutoDock.

While it is clear the above compounds are effective inhibitors of ToxT, in order to better understand the molecular basis of inhibition and elucidate the specific residues and interactions necessary for binding, we used the computational docking program AutoDock^36^ to predict how the compounds bind to ToxT. No significant differences in the free energies of binding were predicted, although there were slight modifications to the orientation of the docked ligands (Supplementary Fig. S3). In order to test the validity of the AutoDock models, we solved the crystal structures of ToxT bound to two of the most promising compounds, **5a** and **3b**, to 2.3 Å and 2.0 Å, respectively. Electron density was visible in the effector-binding pocket of both structures that could not accommodate palmitoleic, oleic, or palmitic acids, but clearly fit the synthetic inhibitors (Fig. 6a and Supplementary Fig. S4). The positions and conformations of the inhibitors bound to ToxT in the X-ray crystal structures were similar to those predicted by AutoDock (Fig. 6c), providing additional confidence in the accuracy of the computational models for compounds for which we did not solve the X-ray structures. In the two crystal structures and all of the AutoDock structures, the compounds bound similarly to cis-palmitoleic acid (Fig. 6b and Supplementary Fig. S3), with the position of the carboxylate varying by 0.02–0.87 Å, suggesting favorable electrostatic interactions between the carboxylate head groups and Lys31 and Lys230. It seems that the bound conformation is dictated primarily by these carboxylate-lysine interactions. Beyond this, the planarity of the compound is the only other obvious factor determining how the inhibitors bind ToxT. For the more planar naphthalene compounds, the aromatic rings sit towards the bottom of the pocket (Fig. 6b, left and Supplementary Fig. S3, top and middle rows). The less planar tetralin rings, in contrast, are rotated clockwise 90°, so the aromatic rings sit towards the left side of the pocket. In this orientation, the fatty acid chain traces the perimeter of the tetralin compound almost perfectly (Fig. 6b, right and Supplementary Fig. S3, bottom row).

### Inhibitors bind ToxT more tightly than virstatin

**Figure 7:**
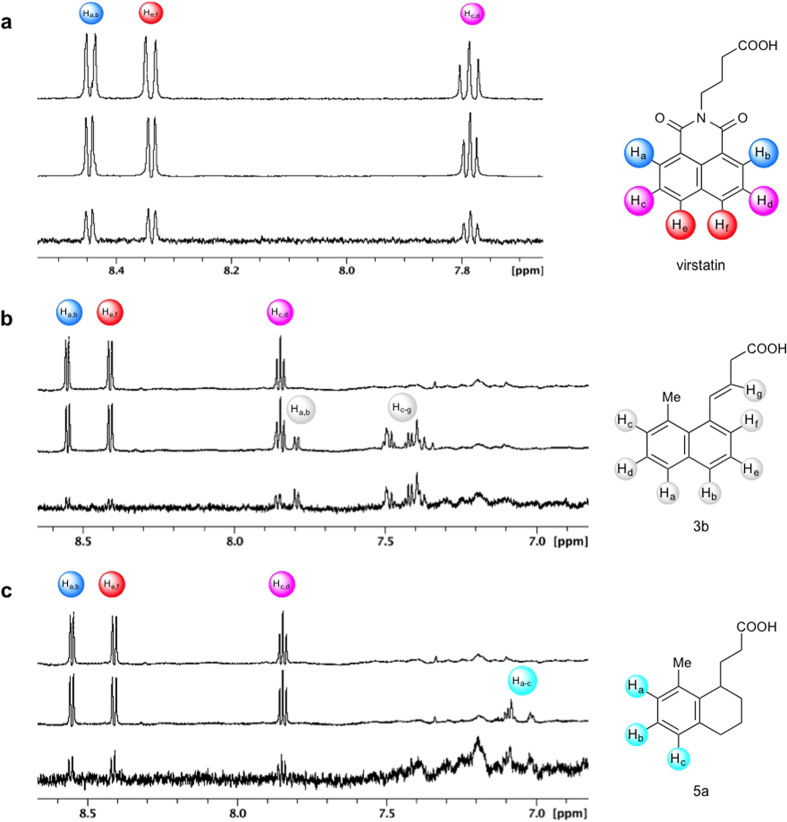
STD NMR verifies direct binding of virstatin to ToxT and competition for the effector-binding pocket between virstatin and compounds 5a and 3b. (**a**) ^1^H-NMR spectrum of virstatin alone (top) and in the presence of 20 μM ToxT (middle). The corresponding STD-difference spectrum (bottom). (**b**,**c**) ^1^H-NMR spectrum of 100 μM virstatin in the presence of 20 μM ToxT (top) and with the addition of compound 3b (**b**, middle) or compound 5a (**c**, middle). Competition STD-difference spectrum showing the reduction in STD signal intensities of virstatin due to competitors 3b (**b**, bottom) and 5a (**c**, bottom). Structures of virstatin, 3b, and 5a with aromatic protons labeled shown to the right of each corresponding spectrum.

Saturation transfer difference (STD) NMR^37^ was used to characterize the strength of ToxT-ligand binding interactions. Significant STD signal was observed for virstatin, confirming it binds to ToxT. As shown in Fig. 7a, saturation is transferred to the aromatic protons of virstatin upon binding to the protein. The STD effect on the aliphatic protons of virstatin was not analyzed due to their spectral overlap with ToxT. Based on the STD spectra for the aromatic protons H_a,b_ and H_e,f_, the K_D_ of virstatin was determined to be 483 ± 109 μM and 331 ± 65 μM, respectively (Supplementary Fig. S5). The relative binding affinities of compounds **3b** and **5a** were determined by competition STD NMR experiments. The compounds were titrated into samples containing 20 μM ToxT and 100 μM virstatin, causing a significant decrease in the STD signals of virstatin (Fig. 7b,c). X-ray crystallography has confirmed the binding pocket of compounds **3b** and **5a**; the decrease in the STD signal of virstatin upon the addition of the competitors suggests virstatin binds in the same effector-binding pocket of ToxT. Of course, an allosteric effect is also possible, with virstatin binding elsewhere on ToxT and influencing ligand binding at the UFA pocket^28^. Based on the calculated K_D_ of virstatin and the decrease in the STD signal, the calculated K_i_ values of **3b** and **5a** were 10 μM and 31 μM, respectively. Despite a suggested tendency for virstatin to bind non-specifically at high concentrations (see Methods), the relative magnitudes of these binding constants are consistent with the activity assays described above.

### Conclusion and Perspectives

The search for a strategy to combat *Vibrio cholerae* infections has been an ongoing focus of research in the field. Bile, unsaturated fatty acids (UFAs), and the small molecule virstatin are among the natural and synthetic inhibitors of *V. cholerae* virulence and its transcriptional activator ToxT^17^,^18^,^19^,^20^,^23^. New targets for the master virulence regulator ToxT have recently been identified^38^, however many are additional fatty acids^21^,^22^. Others include theoretical inhibitors identified computationally^39^, and effectors of *toxT* transcription that fail to inhibit colonization^40^. While unsaturated fatty acids are effective, *V. cholerae* virulence inhibitors, the number of UFAs is finite and the likelihood of identifying an UFA that is significantly more potent than the rest is low. Additionally, what can be learned about ToxT and the detailed mechanisms of virulence inhibition is limited if analyses do not look beyond a single class of natural products. Screening of large small-molecule libraries has identified ToxT inhibitors such as virstatin^23^, as well as others yet to be validated experimentally^39^. While virstatin has been demonstrated to be effective at reducing the transcriptional activity of ToxT *in vitro* and *in vivo*, it has significant limitations. For example, high concentrations were needed to inhibit colonization of the infant mouse (3.5 mM virstatin in the inoculum and 17.65 mM virstatin boosts)^23^. Also, *V. cholerae* strains resistance to virstatin have been identified^25^. As it is thought that virstatin inhibits ToxT activity by preventing dimerization^24^, some non-O1/non-O139 *V. cholerae* strains that demonstrate a stronger ToxT dimerization capability have been shown to be virstatin resistant^25^.

Therefore, in order to inhibit the activity of ToxT, our approach in this work was to design small molecules based on the conformation of the ToxT ligand found in the X-ray crystal structure, *cis*-palmitoleic acid^20^,^26^. As predicted, these “pre-folded” small molecules bind much more tightly than UFAs, which show no activity at the tested concentrations. The compounds do not inhibit colony formation, and are therefore not bactericidal, but were shown to inhibit virulence gene production via β-galactosidase and autoagglutination assays, and ToxT-DNA binding via EMSAs. The compounds bind to the effector-binding pocket of ToxT as predicted by AutoDock and visualized by X-ray crystallography. Based on a calculated K_D_ determined by STD NMR, the lead compounds have at least 10-fold stronger binding affinities than the best-known ToxT inhibitor, virstatin^23^,^24^.

STD NMR data indicate virstatin binds in the same ligand-binding pocket of ToxT, which can accommodate only one ligand at a time. As verified in the X-ray crystal structures of the ToxT-inhibitor complexes, our synthetic compounds bind in the effector-binding pocket, and UFA is no longer observed. Ligand binding, which occurs at the interface between N- and C-terminal domains, clearly inhibits protein-DNA binding (via the C-terminal domain) and may inhibit dimerization (via the N-terminal domain)^33^,^41^,^42^. We will test the effects of compound binding on ToxT dimerization in future studies.

Previously identified *Vibrio cholerae* inhibitors, including virstatin, were identified from large small-molecule libraries via high-throughput screening^23^,^40^. In contrast, we have successfully designed, synthesized, and begun characterization of specific inhibitors of *V. cholerae* ToxT. Our structure-based approach to drug design has resulted not only in a set of small molecules that appear to be the most effective ToxT inhibitors to date, but gives us a general template for designing more potent virulence inhibitors; inherent to our design is potential for a wide degree of variation to improve activity, specificity, and/or bioavailability. These novel compounds, while maintaining an overall folded fatty acid-like shape, have a more constrained conformation that results in tighter binding to ToxT, presumably due to a more favorable entropic contribution to the free energy of binding. Optimization of the degree of saturation of the rings – from aromatic to partially or completely reduced – should allow for better accommodation of the compounds in the non-planar binding pocket. Similarly, varying the length of the carboxylate chain and the substituent groups at the 8 position (i.e. the methyl) could fine-tune solubility, hydrophobic interactions, and further strengthen binding affinities.

The findings presented here describe the design and synthesis of antivirulence compounds specifically targeted to inhibit ToxT, which can potentially serve as novel and effective therapeutics against cholera. Furthermore, as UFAs have been shown to also inhibit other members of the AraC family that regulate virulence gene production^43^, we hypothesize these compounds may be effective at inhibiting virulence gene expression in a variety of bacterial pathogens, and we hope to expand their therapeutic potential in order to advance the treatment and prevention of additional enteric diarrheal diseases.

## Methods

### β-galactosidase Assays

Cultures of the *V. cholerae* O395 *tcpA-lacZ* (MBN135) and *ΔToxT tcpA-lacZ* (MBN142) fusion constructs^44^ were grown for 14 hours with shaking in virulence inducing conditions (LB media pH 6.5 at 30 °C)^8^,^45^. For testing inhibitors, the compounds or DMSO were added to a final concentration of 0.05–50 μM at the time cultures were inoculated. β-galactosidase activity was quantitatively measured according to Miller^46^.

### Western Blot

Whole-cell extracts were assayed for total protein concentration using the Pierce BCA Protein Assay Kit (Thermo Scientific). Samples were subjected to SDS-PAGE on 16% Tris-Glycine gels (Invitrogen) and transferred to a nitrocellulose membrane using iBlot (Invitrogen). The membrane was blocked with 3% bovine serum albumin in TBST (1x Tris-buffered saline, 0.1% Tween), incubated with anti-TcpA antibody^47^, and washed in TBST. After incubation with horseradish peroxidase-conjugated secondary antibody (Bio-Rad), the membrane was washed in TBS. Blots were visualized using the Pierce ECL detection system (Thermo Scientific) according to the manufacturer’s protocols.

### Colony Formation Units Assay

Samples grown in the presence or absence of the compounds were serially diluted and plated on LB-agar plates. Plates were incubated overnight at 37 °C and CFUs were counted. CFUs of cultures grown in the presence of compounds were compared to that of the wild-type O395 culture.

### Autoagglutination Assays

Cultures of the *V. cholerae* classical strain O395 and O395*ΔtoxT* were grown for 14 hours with shaking in inducing conditions (LB media pH 6.5 at 30 °C). The compounds or DMSO were added at the time cultures were inoculated. After 14 hours, cultures were placed at room temperature and observed immediately.

### ToxT Expression and Purification

ToxT was expressed by autoinduction from *toxT-intein/CBD* (chitin binding domain) fusion construct transformed in BL21-CodonPlus (DE3)-RIL *E. coli*, as described previously^20^. Cells were harvested by centrifugation, resuspended in medium-salt column buffer (20 mM Tris, 1 mM EDTA, 500 mM NaCl, pH 7.5), lysed via sonication, and clarified by centrifugation. Clarified supernatant was loaded onto a gravity flow column packed with chitin beads (New England Biolabs) equilibrated in column buffer. After elution of the supernatant, the column was washed with column buffer followed by low-salt buffer (20 mM Tris, 1 mM EDTA, 200 mM NaCl, pH 7.5), and equilibrated with cleavage buffer (low-salt buffer with 100 mM dithiothreitol (DTT)). The column was placed at 4 °C for 16 hours to cleave the intein/CBD. ToxT-intein/CBD fusion protein that co-eluted with the cleaved ToxT was separated using a HiTrap sepharose packed fast flow cationic exchange column (GE) with the following gradient: 45% high-salt buffer (20 mM Tris, 1 mM EDTA, 1 M NaCl, pH 7.5) for 175 minutes at a flow rate of 0.4 ml/min.

### Electrophoretic Mobility Shift Assays

An 84-bp *tcpA* promoter fragment was amplified from *V. cholerae* O395 chromosomal DNA by PCR using 5′ labeled digoxigenin (DIG) primers: DIG-TCP-5 (5′ TGTTTCTTTCA ATGCAAGTG) and DIG-TCP-6 (5′ CACAAAGTCACCTACAATTG). Purified ToxT protein was mixed with 0.5 ng DIG-DNA in a binding buffer (10 mM Tris pH 7.5, 1 mM EDTA, 100 mM KCl, 5 mM MgCl_2_, 1 mM DTT, 0.3 mg/ml BSA, 0.25 μg poly [d(I-C)], and 10% glycerol). Compounds in DMSO were added to a final concentration of 0.1–100 μM, using the same volume of DMSO as a control. To show specificity, 100-fold excess of specific unlabeled dsDNA (84-bp *tcpA* promoter fragment) was added to one reaction tube. Similarly, 0.5 ng non-specific labeled dsDNA (mutated *tcpA* promoter fragment CJ2.6^35^) was added in place of specific DIG-DNA for another. Reactions were incubated for 15 min at 30 °C, loaded on a 5% polyacrylamide gel (1x Tris-Borate EDTA, pH 8), and subjected to electrophoresis in 0.75x TBE at 4 °C. The DNA was transferred onto a positively charged nylon membrane (Roche) by electroblotting using 0.5x TBE at 4 °C, probed with anti-DIG-AP antibody, and visualized by chemiluminescence (Roche).

### Crystallization

ToxT was co-crystallized with the synthesized compounds in hanging drops containing 50% protein buffer (20 mM Tris, 1 mM EDTA, 320 mM NaCl, pH 7.5) and 50% reservoir solution (0.1 M MES pH 6.5 and 15% (w/v) PEG 400). The compound was added to 1.47 mg/ml ToxT at a 20:1 molar excess, and the complex was incubated at 30 °C for 15 minutes before setting up drops. The cryoprotectant for ToxT crystals contained 0.1 M MES pH 6.5, 18% (w/v) PEG 400, and 30% 1,4-butanediol or glycerol.

### X-ray data collection, structure solution, and refinement

Data sets were collected remotely at beam line GM/CA-XSD 23-ID-B at the Advanced Light Source at Argonne National Laboratory. Diffraction data were indexed and integrated with X-ray Detector Software (XDS)^48^ and merged using Phenix^49^. Molecular replacement solutions were obtained with Phenix Phaser-MR^50^ using ToxT (PDB ID 3GBG) with PAM deleted from the PDB. The initial model was built via Phenix AutoBuild^51^. Ligands were built from their SMILES strings using Phenix eLBOW^52^. The corresponding CIF files were viewed and edited in Phenix REEL^52^. Ligands were manually placed into the electron density using Coot^53^,^54^ and visualized using Pymol^55^. Iterative model refinement was performed using Phenix and Coot. Molecules of MES buffer were positioned in a similar manner as the ligands. Data collection and refinement statistics are presented in Supplementary Table S1.

### Molecular Docking

Computational screening was carried out using Autodock 4.0.1^36^. The coordinates for the receptor were obtained from the Protein Data Bank (PDB ID 3GBG) and were modified to exclude the bound PAM. The ligands were prepared from their SMILES description in Chimera^56^. All ligands as well as CD_CE and CE_NZ of lysine residues 230 and 31 of the receptor were considered flexible. A grid box with 35 × 40 × 32 points and a grid point spacing of 0.375 Å (gridcenter 54.5 × 46.5 × 20) encompassed the known binding pocket. Each docking simulation involved 20 evaluations using the Lamarkian genetic algorithm.

### STD NMR

#### Sample preparation

Virstatin was solubilized in DMSO-d_6_ and compounds **5a** and **3b** in DMSO, at working stock concentrations of 35.5 mM, 394 mM, and 50.5 mM, respectively. Purified ToxT in protein buffer (20 mM Tris, 1 mM EDTA, 320 mM NaCl, pH 7.5) was used at a final concentration of 20 μM. All samples contained 50 μM 3-(Trimethylsilyl)-1-propanesulfonic acid sodium salt (TSP) as an internal standard and 5% D_2_O. All NMR experiments were carried out on a Bruker Avance 600 MHz or 700 MHz spectrometer equipped with a TCI cryogenic probe. Samples were stored at 4 °C prior to acquisition.

#### Determination of K_D_ of virstatin

K_D_ determination followed the protocol outlined by Angulo *et al*.^57^. For on-resonance spectra, the protein was saturated at 500 Hz or 583.33 Hz for the 600 MHz and 700 MHz NMR spectrometers, respectively, for 1–4 seconds utilizing a train of 50 ms Gaussian pulses. For off-resonance spectra, the sample was irradiated at −2000 Hz. The total relaxation delay was 7 seconds. Data acquisition consisted of 128 scans and 32,768 points. The STD-effect was calculated by measuring the intensity of the virstatin aromatic proton peaks in the on- and off-resonance spectra:


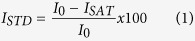


The STD amplification factor (A_STD_) values for different protons were fitted by using^37^:


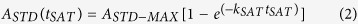


where *A*_*STD*_ = *I*_*STD*_ *ligand excess*.

The initial slopes STD-AF_0_ (the initial growth rates of the STD amplification factors, which correspond to A_STD_ at zero t_SAT_) are obtained from:


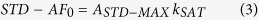


where A_STD-MAX_ is the maximal achievable A_STD_ (for a very long saturation time).

The K_D_ of virstatin was calculated using Michaelis-Menten kinetics. The STD-AF_0_ was plotted as a function of the ligand concentration to construct the binding (Langmuir) isotherm:


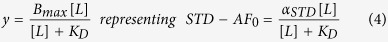


We determined the binding affinity of virstatin for ToxT in a concentration range of 66.5 to 665 μM, collecting data for 7 different concentrations of virstatin. At concentrations higher than 665 μM, we observed a perturbation of the STD-effect attributable to non-specific binding of virstatin. It has been previously observed that non-specific binding at high ligand concentrations and high ligand/protein ratios can interfere with STD experiments, since the non-specific binding can contribute to the total STD-effect^58^,^59^. We therefore did not include data points higher than 665 μM.

#### Competition studies between virstatin and compounds 5a and 3b

Virstatin was used as the STD indicator at a concentration of 100 μM. Compounds **5a** and **3b** were added at concentrations of 20–200 μM. STD spectra were obtained as above, with a saturation time of 3 seconds. The calculation of the competitive inhibition was as follows^60^:


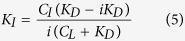


where C_I_ is the concentration of inhibitor with unknown K_D_ (compound **5a** or **3b**), C_L_ is the concentration of ligand with known K_D_ (virstatin), K_D_ is that of virstatin, and i is the inhibition expressed as a fraction: 
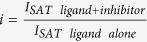
.

## Additional Information

**How to cite this article:** Woodbrey, A. K. *et al*. A new class of inhibitors of the AraC family virulence regulator *Vibrio cholerae* ToxT. *Sci. Rep.* **7**, 45011; doi: 10.1038/srep45011 (2017).

**Publisher's note:** Springer Nature remains neutral with regard to jurisdictional claims in published maps and institutional affiliations.

## Supplementary Material

Supplementary Information
