# Supplementary material for: A new class of inhibitors of the AraC family virulence regulator Vibrio cholerae ToxT
Source: Sci Rep. 2017 Mar 23;7:45011. doi: 10.1038/srep45011 (PMC5362913; doi:10.1038/srep45011)
Supplement: Supplementary Information [file srep45011-s1.pdf]

Supplementary Information

A new class of inhibitors of the AraC family virulence regulator *Vibrio cholerae* ToxT

Anne K. Woodbrey[1], Evans O. Onyango[1], Maria Pellegrini[1], Gabriela Kovacikova[2], Ronald K. Taylor[2], Gordon W. Gribble[1], F. Jon Kull[1]*

[1] Department of Chemistry, Dartmouth College, Hanover, NH, 03755, USA
[2] Department of Microbiology and Immunology, Geisel School of Medicine at Dartmouth, Hanover, NH, 03755, USA
* F.Jon.Kull@dartmouth.edu

Contents

Supplementary Figures S1 – S5	p. 2-6
Supplementary Table S1	p. 7
Supplementary Experimental Procedures	p. 8-12
Experimental Spectra of synthesized compounds	p. 13-22

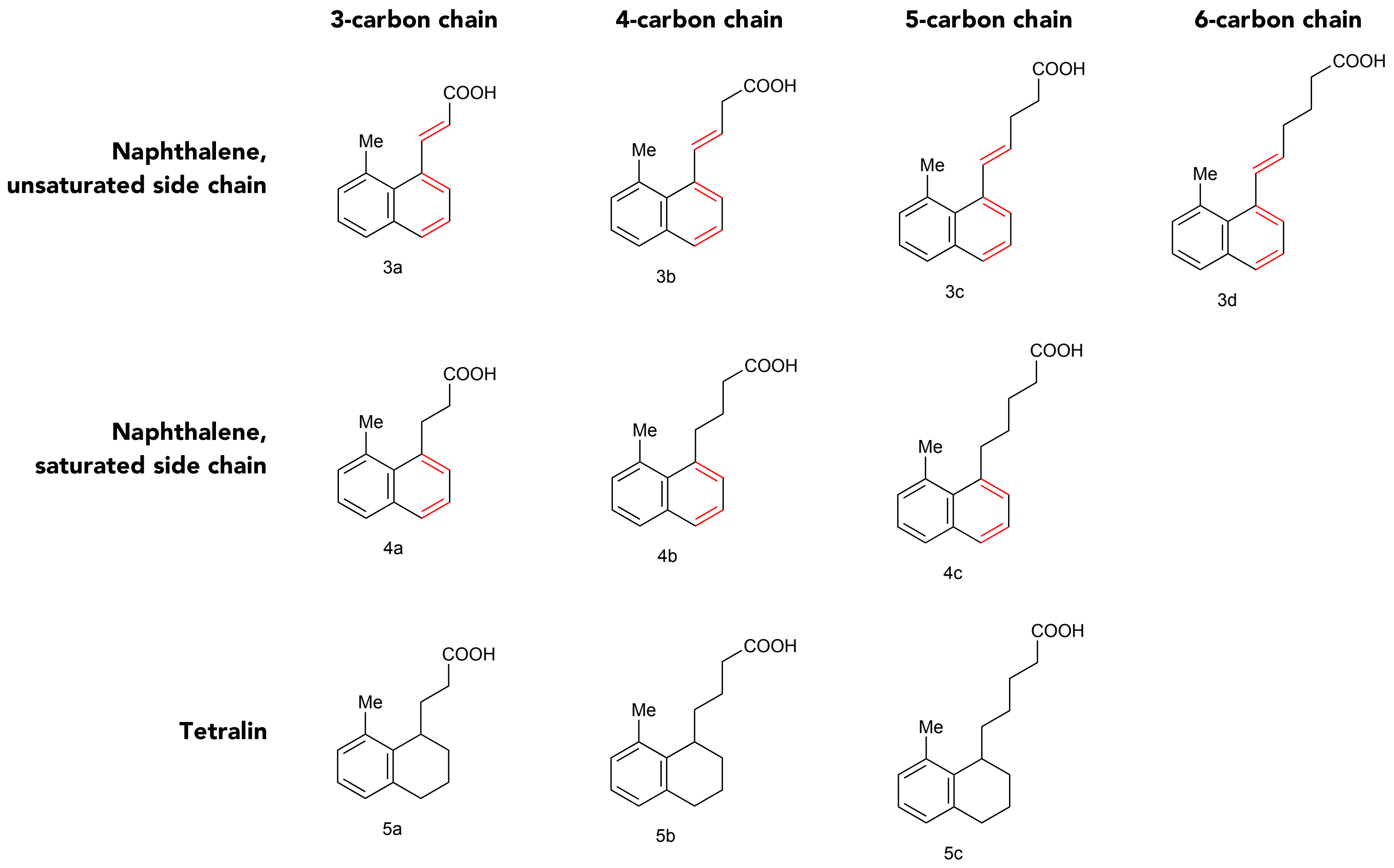

Supplementary Figure S1. Structures of the ten synthesized compounds. The carboxylate chain varies by the number of carbon atoms (3-6) and level of saturation (monounsaturated or fully saturated). The saturation of the right-handed ring also varies (naphthalene or tetralin). Compounds 3a-d (naphthalenes, unsaturated side chain) are the E isomer. The stereochemistry of the tetralins is undefined, since these compounds are racemic.

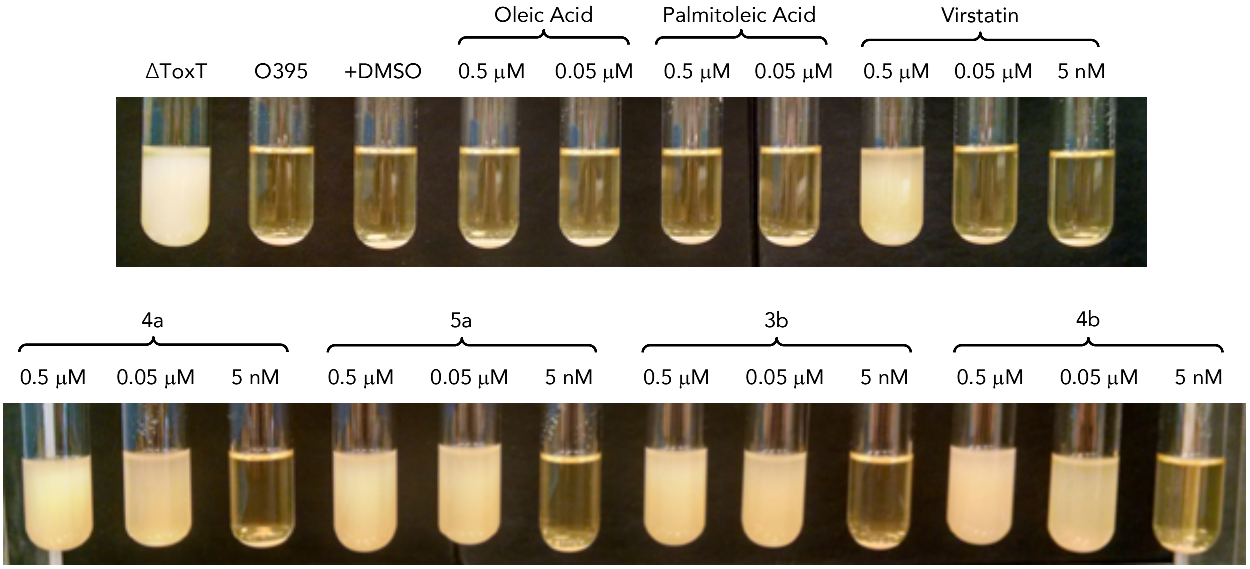

Supplementary Figure S2. Unsaturated fatty acids (UFAs) do not inhibit *V. cholerae* autoagglutination at low concentrations. Autoagglutination of O395 cultures grown in the presence of 0.5 μM, 0.05 μM, and 5 nM virstatin and compounds 4a, 5a, 3b, and 4b. Cultures containing 0.5 μM and 0.05 μM oleic and palmitoleic acids were grown similarly; the UFAs were not soluble at 5 nM.

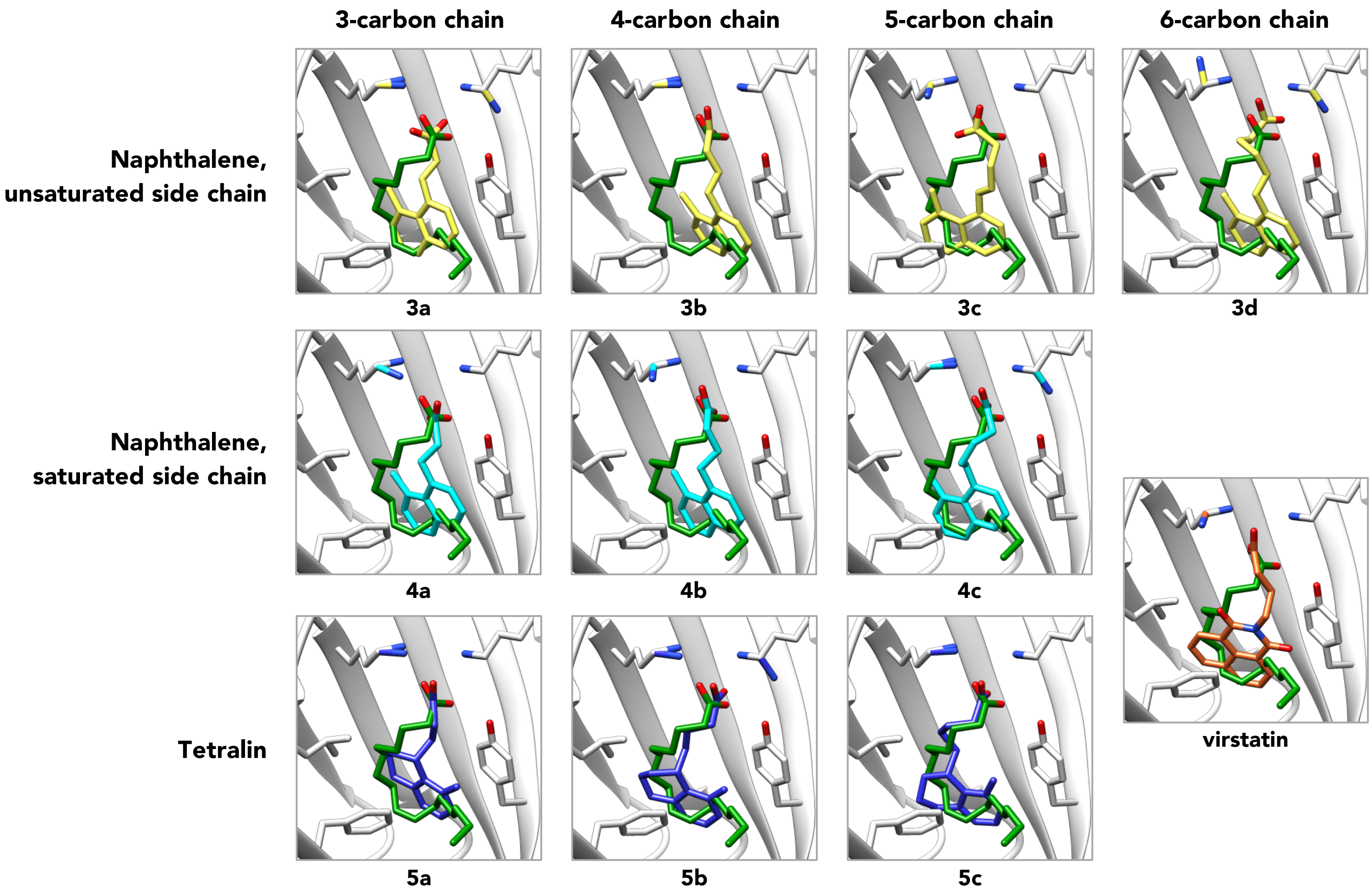

Supplementary Figure S3. The fatty acid binding region of ToxT comparing *cis*-palmitoleate (green, from crystal structure 3GBG) and models of each compound. The naphthalenes with a double bond in the carboxylate chain are yellow, the naphthalenes with a single bond are cyan, tetralins are dark blue, and virstatin is orange. Bound conformations predicted using Autodock. Figures made with Chimera.

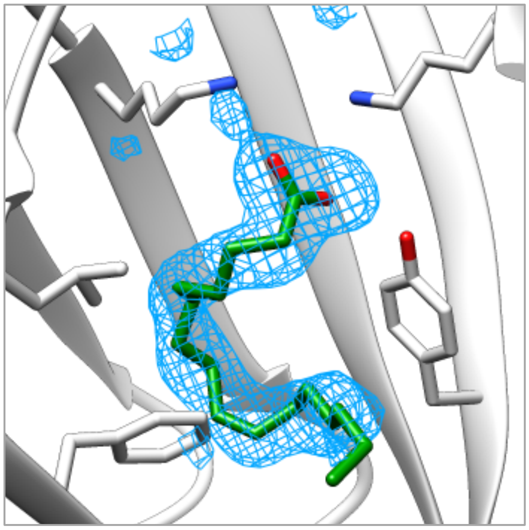

Supplementary Figure S4. Electron density map of the natural fatty acid ligand. Simulated annealing $F_o - F_c$ omit map of ToxT bound to *cis*-palmitoleate contoured at 2.5 σ (PDB 3GBG).

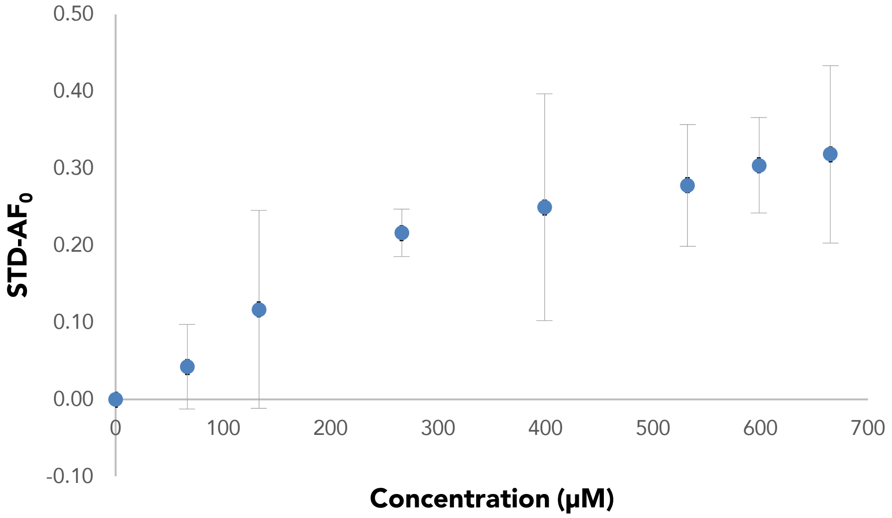

Supplementary Figure S5. STD-AF$_0$ curve of virstatin as a function of ligand concentration. Data were fitted to equation (3) (see Methods). The isotherm was constructed using the STD data from protons $H_{e,f}$ of virstatin. The same was done for protons $H_{a,b}$ (data not shown). Error bars are standard deviation calculated via error propagation.

Supplementary Table S1. Crystallographic Data Collection and Refinement Statistics

	ToxT-A77	ToxT-A78
**Data collection**		
Space group	$P2_12_12_1$	$P2_12_12_1$
Cell dimensions		
a, b, c (Å)	40.31, 81.11, 83.85	40.52, 80.96, 84.49
α, β, γ (°)	90, 90, 90	90, 90, 90
Resolution (Å)	41.93 - 2.30 (2.40 - 2.30)	42.25 - 2.00 (2.10 - 2.00)
R_{sym} or R_{merge}	0.138 (1.11)	0.088 (0.811)
I / σI	11.35 (2.68)	11.91 (2.03)
CC(1/2)	0.997 (0.810)	0.998 (0.663)
Completeness (%)	100 (100)	99.7 (99.8)
Redundancy	7.1 (7.3)	4.1 (4.1)
**Refinement**		
Resolution (Å)	41.93 - 2.30	42.25 - 2.00
No. reflections (unique)	12,783 (1508)	19,368 (2564)
R_{work} / R_{free}	0.169/0.230	0.189/0.230
No. atoms		
Protein	2,204	2,191
Compound	16	17
Ligand/ion	24	24
Water	67	79
B-factors		
Protein	49.33	42.35
Ligand/ion	67.39	56.42
Water	45.76	41.74
R.m.s. deviations		
Bond lengths (Å)	0.007	0.003
Bond angles (°)	0.854	0.611

Data were collected from one crystal for each structure. Data collection values obtained from XDS. Refinement values obtained from PDB.

*Values in parentheses are for highest-resolution shell.

Supplementary Experimental Procedures

General Methods for Synthetic Procedures

All reactions were conducted in oven-dried glassware under nitrogen gas (N_2). Solvents were reagent grade. All reagents were obtained commercially as reagent grade and, unless otherwise noted, used without further purification. Reactions were monitored by TLC silica gel plates (0.25 mm) and visualized by UV light or p-anisaldehyde. Column chromatography was performed using silica gel (60, particle size 40-60 mm). ^{1}H NMR data were recorded on a Bruker Avance III 500 MHz spectrometer (TBI probe) and a Bruker Avance III 600 MHz spectrometer (BBFO probe) with calibration of spectra to $CDCl_3$ (7.26 ppm) or CH_2Cl_2 (5.32 ppm). ^{13}C NMR data were recorded at 150 MHz on the 600 MHz spectrometer with calibration of spectra to the central line of $CDCl_3$ (77.16 ppm) or CH_2Cl_2 (53.84 ppm). High-resolution mass spectra (HRMS) were obtained employing electron ionization (EI), with TOF as the mass analyzer.

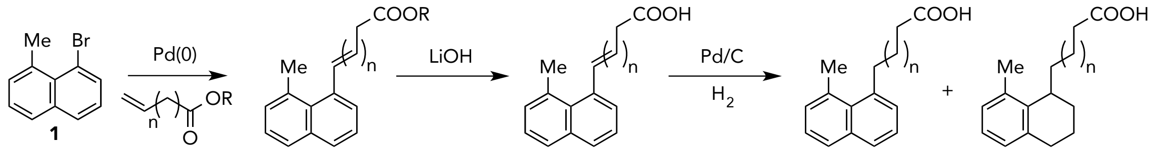

1

R= H, Me, or Et
n= 0-3
57-86%

2

3

4

5

Supplementary Figure S6. General synthetic scheme of small-molecule inhibitors.
Supplementary Fig. S6 is identical to Fig. 3 but is shown again for easy referencing.

General procedure 1 (Heck coupling)

To a solution of 1-bromo-8-methylnaphthalene (**1**) (1 eq.), palladium(II) acetate (0.05 eq.), tri-*o*-tolylphosphine (0.1 eq.), and triethylamine (10 eq.) in DMF was added alkene (3.5 eq.) dropwise. The reaction mixture was stirred overnight at 110°C, then cooled to room temperature and quenched with water. The organic layer was extracted twice with EtOAc, washed with brine, dried over anhydrous $MgSO_4$, filtered, and concentrated *in vacuo*. Purification of the crude mixture via silica gel chromatography and in some cases recrystallization/trituration yielded the corresponding product **2**.

General procedure 2 (ester hydrolysis)

To a solution of **2** (1 eq.) in MeOH/H_20 (3:1) was added LiOH (3 eq.). The mixture was stirred overnight at room temperature. The solution was partially concentrated *in vacuo* and quenched with 1M HCl. The organic layer was extracted with EtOAc, washed with brine, dried over anhydrous $MgSO_4$, filtered, and concentrated *in vacuo*. The crude product was recrystallized and/or triturated to yield the acid **3**.

General procedure 3 (hydrogenation)

To a solution of **2** or **3** (1 eq.) in CH_2Cl_2/MeOH (1:1) was added 10% Pd-C (0.1 eq.). The mixture was stirred vigorously at room temperature under H_2 for 30 minutes. The mixture was filtered through Celite and concentrated *in vacuo*. Trituration and/or recrystallization resulted in **4** and trace amounts of **5** (or their ester equivalents). For reactions allowed to proceed overnight, only **5** was obtained. In some cases, **5** was conveniently obtained using **4** as SM.

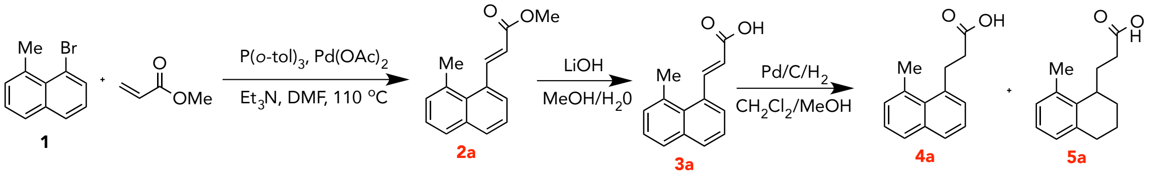

Supplementary Figure S7. Synthesis of compounds 3a, 4a, and 5a. Based on Supplementary Fig. S6.

(*E*)-3-(8-Methylnaphthalen-1-yl)acrylic acid (3a)

General procedure 1 was followed using **1** (60 mg, 0.271 mmol), $Pd(OAc)_2$ (6.09 mg, 0.0271 mmol), P(*o*-tol)$_3$ (16.52 mg, 0.0543 mmol), Et_3N (0.38 mL, 2.714 mmol), DMF (0.1M, 3 mL), and methyl acrylate (0.09 mL, 0.950 mmol). The crude mixture, a green-brown oil, was purified via silica gel chromatography (20:1 hexanes/ethyl acetate) to afford **2a** (53 mg, 86%) as colorless oil. General procedure 2 was followed using **2a** (53 mg, 0.234 mmol), MeOH/H_20 (3:1; 3 mL), and LiOH (29.48 mg, 0.703 mmol) to yield **3a** as a white solid (48 mg, 97%), which was further purified by trituration with hexanes. TLC (hexanes:EtOAc, 3:1 v/v): R_f = 0.30; ^{1}H NMR (500 MHz, $CDCl_3$): δ 8.82 (d, *J* = 15.6 Hz, 1H), 7.87 (dd, *J* = 8.1, 1.1 Hz, 1H), 7.73 (dd, *J* = 7.8, 1.4 Hz, 1H), 7.52 (d, *J* = 7.0 Hz, 1H), 7.45 – 7.36 (m, 3H), 6.24 (d, *J* = 15.5 Hz, 1H), 2.87 (s, 3H); ^{13}C NMR (150 MHz, $CDCl_3$): δ 172.0, 152.0, 135.0, 134.9, 134.3, 131.6, 131.5, 130.4, 128.1, 127.7, 126.1, 125.1, 118.6, 25.7; HRMS (*m/z*): $[M]^+$ calcd. for $C_{14}H_{12}O_2$, 212.0837; found, 212.0841.

3-(8-Methylnaphthalen-1-yl)propanoic acid (4a)

General procedure 3 was followed using **3a** (46.7 mg, 0.220 mmol), CH_2Cl_2/MeOH (1:1; 2.5 mL), and 10% Pd-C (23.03 mg, 0.022 mmol). The crude was triturated with MeOH to yield **4a** as a pure white solid in quantitative yield. (The trace amounts of the tetralin derivative dissolved in MeOH, while the naphthalene did not). TLC (hexanes:EtOAc, 3:1 v/v): R_f = 0.29; ^{1}H NMR (500 MHz, $CDCl_3$): δ 7.75 – 7.70 (m, 2H), 7.38 – 7.29 (m, 4H), 3.66 – 3.62 (m, 2H), 2.93 (s, 3H), 2.73 – 2.70 (m, 2H); ^{13}C NMR (150 MHz, $CDCl_3$): δ 177.8, 137.5, 135.9, 134.2, 132.0, 130.5, 129.0, 128.5, 125.3, 125.2, 37.0, 31.9, 25.7; HRMS (*m/z*): $[M]^+$ calcd. for $C_{14}H_{14}O_2$, 214.0994; found, 214.0999.

3-(8-Methyl-1,2,3,4-tetrahydronaphthalen-1-yl)propanoic acid (5a)

General procedure 3 was followed using **4a** (40 mg, 0.187 mmol), CH_2Cl_2/MeOH (1:1; 2 mL), and 10% Pd-C (19.54 mg, 0.0187 mmol). The reaction yielded **5a** as yellowish oil (39 mg, 96%). TLC (hexanes:EtOAc, 3:1 v/v): R_f = 0.34; ^{1}H NMR (600 MHz, $CDCl_3$): δ 7.04 – 6.91 (m, 3H), 2.82 – 2.74 (m, 3H), 2.55 – 2.41 (m, 2H), 2.31 (s, 3H), 1.93 – 1.67 (m, 6H); ^{13}C NMR (150 MHz, $CDCl_3$): δ 178.1, 139.4, 136.5, 136.1, 128.1, 127.3, 125.8, 34.0, 32.2, 29.4, 28.8, 25.4, 19.1, 17.7; HRMS (*m/z*): $[M]^+$ calcd. for $C_{14}H_{18}O_2$, 218.1307; found, 218.1310.

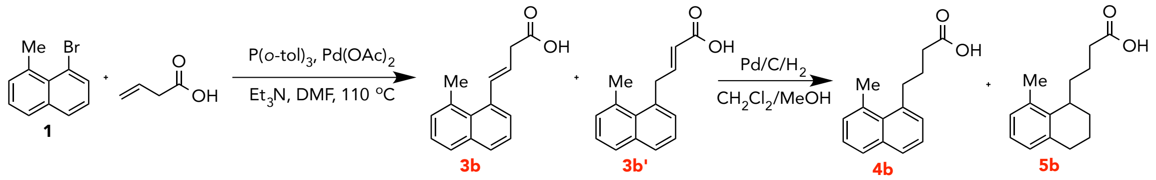

Supplementary Figure S8. Synthesis of compounds 3b, 4b, and 5b. Based on Supplementary Fig. S6.

(*E*)-4-(8-Methylnaphthalen-1-yl)but-3-enoic acid (3b)

General procedure 1 was followed using **1** (100 mg, 0.452 mmol), $Pd(OAc)_2$ (5.08 mg, 0.0226 mmol), P(*o*-tol)$_3$ (13.77 mg, 0.0452 mmol), Et_3N (0.63 mL, 4.52 mmol), DMF (0.1M, 2.5 mL), and but-3-enoic acid (0.13 mL, 1.583 mmol). The crude mixture, an orange oil, was purified via silica gel chromatography (20:1 to 2:1 hexanes/ethyl acetate) to afford a mixture of **3b** and **3b′**. Further purification by trituration with hot hexanes left the pure product **3b** as a white solid (58.7 mg, 57%). TLC (hexanes:EtOAc, 3:1 v/v): R_f = 0.23; ^{1}H NMR (500 MHz, CD_2Cl_2): δ 7.77 – 7.74 (m, 1H), 7.68 (d, *J* = 7.9 Hz, 1H), 7.44 (d, *J* = 15.6 Hz, 1H), 7.39 – 7.27 (m, 4H), 5.97 – 5.90 (m, 1H), 3.37 (dd, *J* = 7.0, 1.5 Hz, 2H), 2.84 (s, 3H); ^{13}C NMR (150 MHz, CD_2Cl_2): δ 175.7, 138.5, 137.6, 135.7, 135.3, 131.7, 129.8, 129.4, 128.2, 127.7, 125.7, 125.5, 122.6, 38.0, 26.0; HRMS (*m/z*): $[M]^+$ calcd. for $C_{15}H_{14}O_2$, 226.0994; found, 226.0995.

*The structure of **3b** was unambiguously assigned based on HMBC NMR analysis.*

4-(8-Methylnaphthalen-1-yl)butanoic acid (4b)

General procedure 3 was followed using a mixture of **3b** and **3b′** (52 mg, 0.230 mmol), CH_2Cl_2/MeOH (1:1; 2.5 mL), and 10% Pd-C (24.05 mg, 0.023 mmol). The crude orange solid was triturated with MeOH to yield **4b** as a pure white solid (50 mg, 96%). TLC (hexanes:EtOAc, 3:1 v/v): R_f = 0.28; ^{1}H NMR (500 MHz, $CDCl_3$): δ 7.72 – 7.69 (m, 2H), 7.36 – 7.26 (m, 4H), 3.31 (t, *J* = 7.8 Hz, 2H), 2.91 (s, 3H), 2.45 (t, *J* = 7.3 Hz, 2H), 2.03 – 1.97 (m, 2H); ^{13}C NMR (150 MHz, $CDCl_3$): δ 178.2, 138.7, 136.0, 134.4, 132.1, 130.3, 129.4, 128.6, 128.4, 125.1, 36.2, 33.4, 28.0, 25.5; HRMS (*m/z*): $[M]^+$ calcd. for $C_{15}H_{16}O_2$, 228.1150; found, 228.1149.

4-(8-Methyl-1,2,3,4-tetrahydronaphthalen-1-yl)butanoic acid (5b)

General procedure 3 was followed using **4b** (45 mg, 0.197 mmol), CH_2Cl_2/MeOH (1:1; 2 mL), and 10% Pd-C (20.63 mg, 0.0197 mmol). The reaction yielded **5b** as colorless oil in quantitative yield. TLC (hexanes:EtOAc, 3:1 v/v): R_f = 0.35; ^{1}H NMR (600 MHz, $CDCl_3$): δ 7.02 – 6.90 (m, 3H), 2.82 – 2.73 (m, 3H), 2.46 – 2.36 (m, 2H), 2.28 (s, 3H), 1.92 – 1.43 (m, 8H); ^{13}C NMR (150 MHz, $CDCl_3$): δ 178.3, 140.2, 136.5, 136.0, 128.1, 127.2, 125.6, 34.6, 33.9, 33.7, 29.6, 25.6, 23.3, 19.1, 17.8; HRMS (*m/z*): $[M]^+$ calcd. for $C_{15}H_{20}O_2$, 232.1463; found, 232.1470.

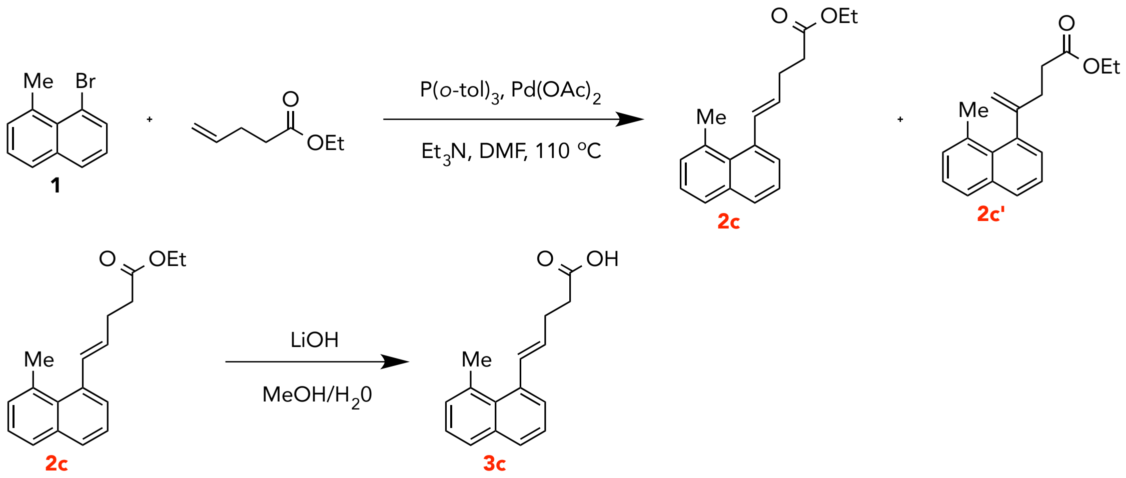

Supplementary Figure S9. Synthesis of compound 3c. Based on Supplementary Fig. S6.

(*E*)-5-(8-Methylnaphthalen-1-yl)pent-4-enoic acid (3c)
General procedure 1 was followed using **1** (1.3 g, 5.88 mmol), $Pd(OAc)_2$ (66.01 mg, 0.294 mmol), P(*o*-tol)$_3$ (179.0 mg, 0.588 mmol), Et_3N (8.17 mL, 58.80 mmol), DMF (0.1M, 60 mL), and ethyl pent-4-enoate (2.93 mL, 20.58 mmol). The crude mixture, a dark brown oil, was purified via silica gel chromatography (50:1 hexanes/ethyl acetate) to afford **2c** (1.05 g, 67%) as orange oil. General procedure 2 was followed using **2c** (100 mg, 0.373 mmol), MeOH/$H_2$0 (3:1; 3 mL), and LiOH (46.91 mg, 1.118 mmol). The crude was triturated with hexanes to yield **3c** as a white solid in quantitative yield. TLC (hexanes:EtOAc, 3:1 v/v): R_f = 0.33; ^{1}H NMR (500 MHz, CD_2Cl_2): δ 7.74 (dd, *J* = 7.6, 1.9 Hz, 1H), 7.68 (d, *J* = 7.9 Hz, 1H), 7.37 – 7.29 (m, 4H), 7.25 (d, *J* = 7.0 Hz, 1H), 5.88 – 5.82 (m, 1H), 2.84 (s, 3H), 2.65 – 2.62 (m, 4H); ^{13}C NMR (150 MHz, $CDCl_3$): δ 179.4, 137.8, 135.8, 135.4, 135.0, 131.5, 129.5, 129.0, 128.9, 127.9, 127.5, 125.4, 125.2, 33.7, 28.2, 26.0; HRMS (*m/z*): $[M]^+$ calcd. for $C_{16}H_{16}O_2$, 240.1150; found, 240.1151.

***2c'** *is identified based on the signature doublet with a large coupling constant around 5.20 ppm.*

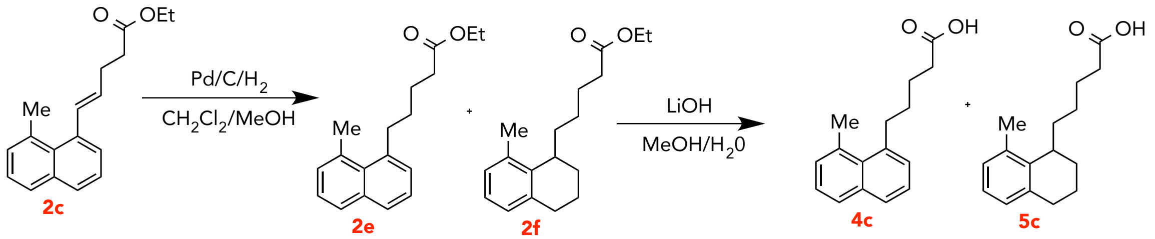

Supplementary Figure S10. Synthesis of compounds 4c and 5c. Based on Supplementary Fig. S6.

5-(8-Methylnaphthalen-1-yl)pentanoic acid (4c)
General procedure 3 was followed using **2c** (106 mg, 0.395 mmol), CH_2Cl_2/MeOH (1:1; 4 mL), and 10% Pd-C (41.34 mg, 0.0395 mmol) to yield **2e** as pale yellow oil (100 mg, 94%), which was used in the next step without further purification. General procedure 2 was followed using **2e** (100 mg, 0.370 mmol), MeOH/$H_2$0 (3:1; 3 mL), and LiOH (46.56 mg, 1.110 mmol) to yield a pale yellow solid (83 mg, 93%). The crude product was recrystallized in MeOH to yield **4c** as a white

solid. TLC (hexanes:EtOAc, 3:1 v/v): R_f = 0.30; ^{1}H NMR (600 MHz, $CDCl_3$): δ 7.70 (d, *J* = 7.7 Hz, 2H), 7.35 – 7.26 (m, 4H), 3.27 (t, *J* = 7.7 Hz, 2H), 2.90 (s, 3H), 2.40 (t, *J* = 7.3 Hz, 2H), 1.82 – 1.76 (m, 2H), 1.73 – 1.69 (m, 2H); ^{13}C NMR (150 MHz, CD_2Cl_2): δ 178.4, 140.1, 136.2, 135.0, 132.4, 130.3, 129.3, 128.5, 128.4, 125.3, 37.0, 33.9, 33.2, 25.6, 24.9; HRMS (*m/z*): $[M]^+$ calcd. for $C_{16}H_{18}O_2$, 242.1307; found, 242.1309.

5-(8-Methyl-1,2,3,4-tetrahydronaphthalen-1-yl)pentanoic acid (5c)

General procedure 3 was followed using **2c** (88 mg, 0.328 mmol), CH_2Cl_2/MeOH (1:1; 3 mL), and 10% Pd-C (34.33 mg, 0.0328 mmol) to yield **2f** as pale yellow oil in quantitative yield, which was used in the next step without further purification. General procedure 2 was followed using **2f** (90 mg, 0.328 mmol), MeOH/H_20 (3:1; 3 mL), and LiOH (41.32 mg, 0.985 mmol) to yield **5c** as pale yellow oil (71 mg, 88%). TLC (hexanes:EtOAc, 3:1 v/v) R_f = 0.43; ^{1}H NMR (500 MHz, CD_2Cl_2): δ 6.98 – 6.86 (m, 3H), 2.79 – 2.70 (m, 3H), 2.41 – 2.35 (m, 2H), 2.27 (s, 3H), 1.87 – 1.39 (m, 10H); ^{13}C NMR (150 MHz, CD_2Cl_2): δ 178.0, 140.9, 136.7, 136.2, 128.1, 127.3, 125.6, 34.9, 34.2, 34.0, 29.8, 27.8, 25.9, 25.2, 19.1, 18.1; HRMS (*m/z*): $[M]^+$ calcd. for $C_{16}H_{22}O_2$, 246.1620; found, 246.1619.

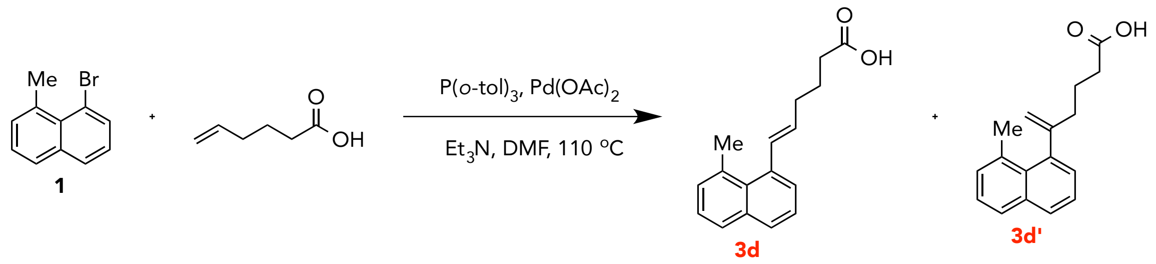

Supplementary Figure S11. Synthesis of compound 3d. Based on Supplementary Fig. S6.

(*E*)-6-(8-Methylnaphthalen-1-yl)hex-5-enoic acid (3d)

General procedure 1 was followed using **1** (100 mg, 0.452 mmol), $Pd(OAc)_2$ (5.08 mg, 0.0226 mmol), P(*o*-tol)$_3$ (13.77 mg, 0.0452 mmol), Et_3N (0.63 mL, 4.52 mmol), DMF (0.1M, 2.5 mL), and hex-5-enoic acid (0.19 mL, 1.583 mmol). The crude mixture, an orange oil, was purified via silica gel chromatography (20:1 to 2:1 hexanes/ethyl acetate) to afford a mixture of hex-5-enoic acid, **3d**, and **3d'** *. Further purification by trituration first with hot hexanes (to remove the hexenoic acid) and second by EtOAc (to remove **3d'**) left the pure product **3d** as a white solid (65.5 mg, 57%). TLC (hexanes:EtOAc, 3:1 v/v): R_f = 0.32; ^{1}H NMR (500 MHz, CD_2Cl_2): δ 7.73 (dd, *J* = 7.3, 2.1 Hz, 1H), 7.67 (d, *J* = 7.9 Hz, 1H), 7.37 – 7.26 (m, 5H), 5.87 – 5.80 (m, 1H), 2.86 (s, 3H), 2.47 (t, *J* = 7.5 Hz, 2H), 2.38 – 2.33 (m, 2H), 1.92 – 1.86 (m, 2H); ^{13}C NMR (150 MHz, CD_2Cl_2): δ 177.8, 138.5, 135.9, 135.5, 135.3, 131.8, 130.9, 129.6, 129.0, 128.0, 127.7, 125.6, 125.5, 33.5, 32.7, 26.2, 24.7; HRMS (*m/z*): $[M]^+$ calcd. for $C_{17}H_{18}O_2$, 254.1307; found, 254.1307.

****3d'** is identified based on the signature doublet with a large coupling constant around 5.2 ppm.*

Experimental Spectra

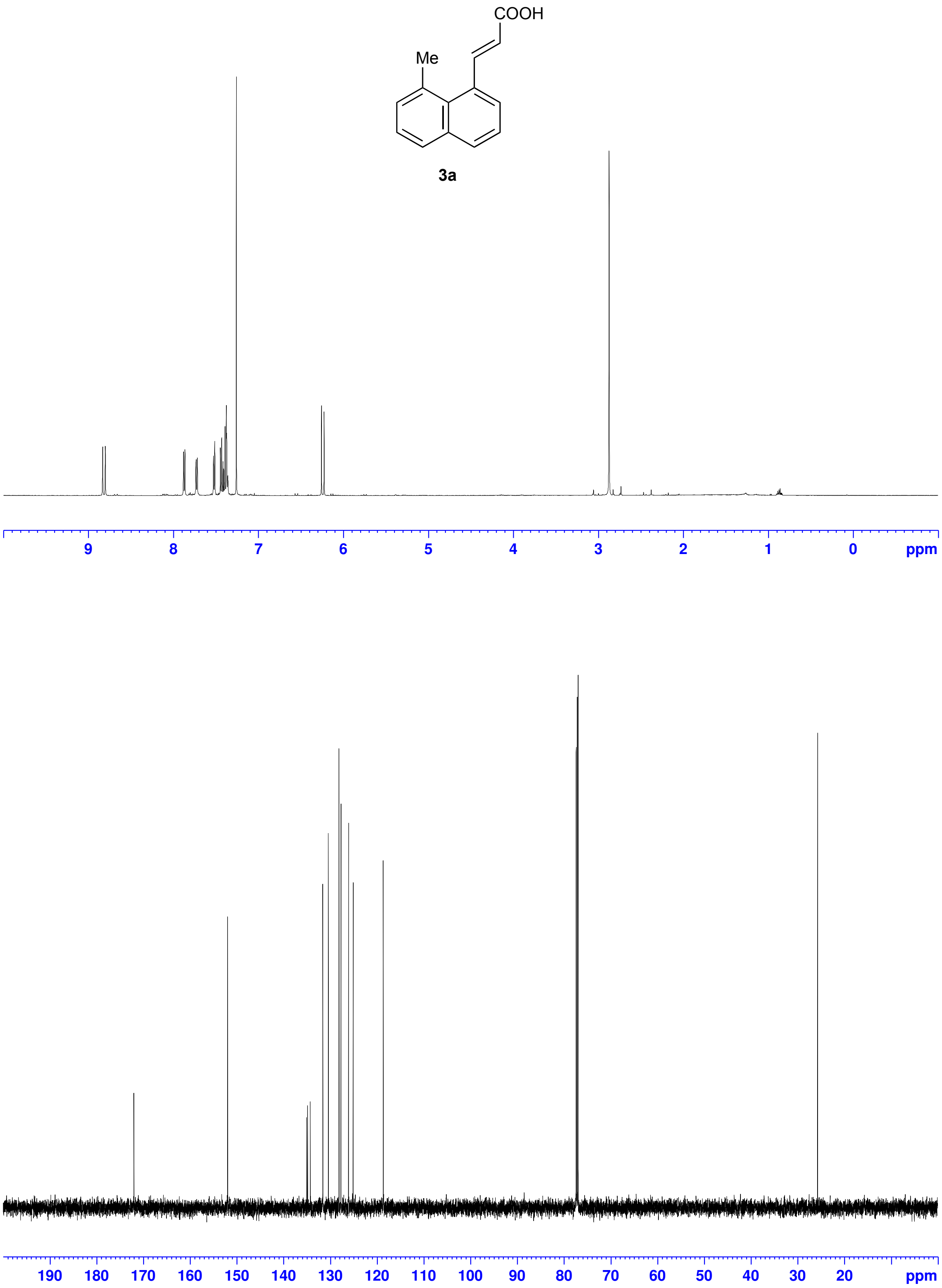

Supplementary Figure S12. ^{1}H NMR (500 MHz, $CDCl_3$) and ^{13}C NMR (150 MHz, $CDCl_3$) of **3a.**

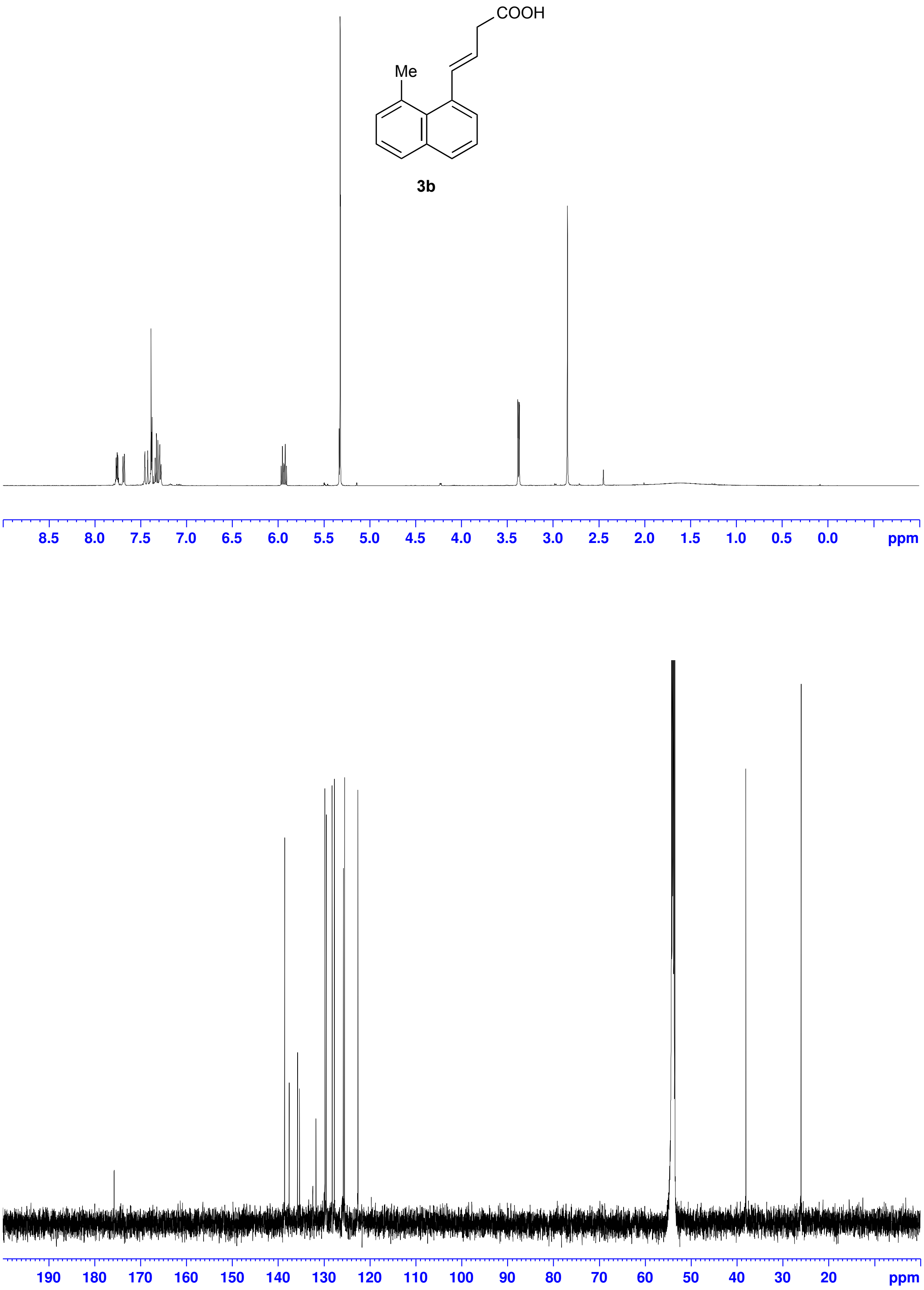

Supplementary Figure S13. ^{1}H NMR (500 MHz, CD_2Cl_2) and ^{13}C NMR (150 MHz, CD_2Cl_2) of **3b.**

3c

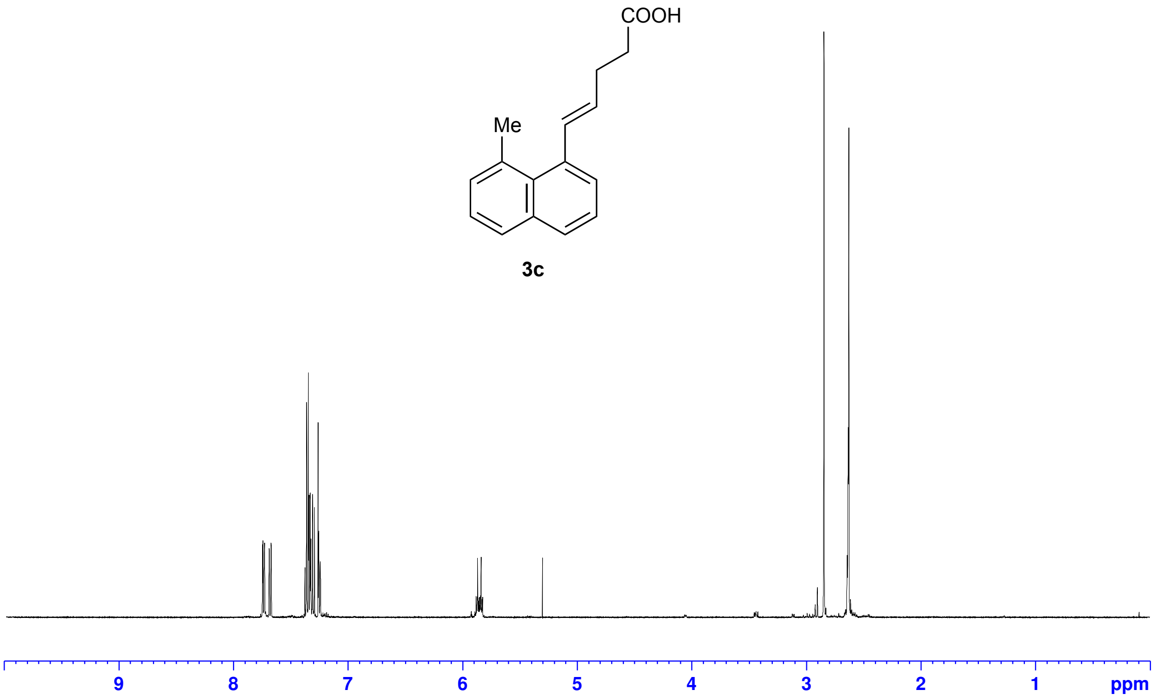

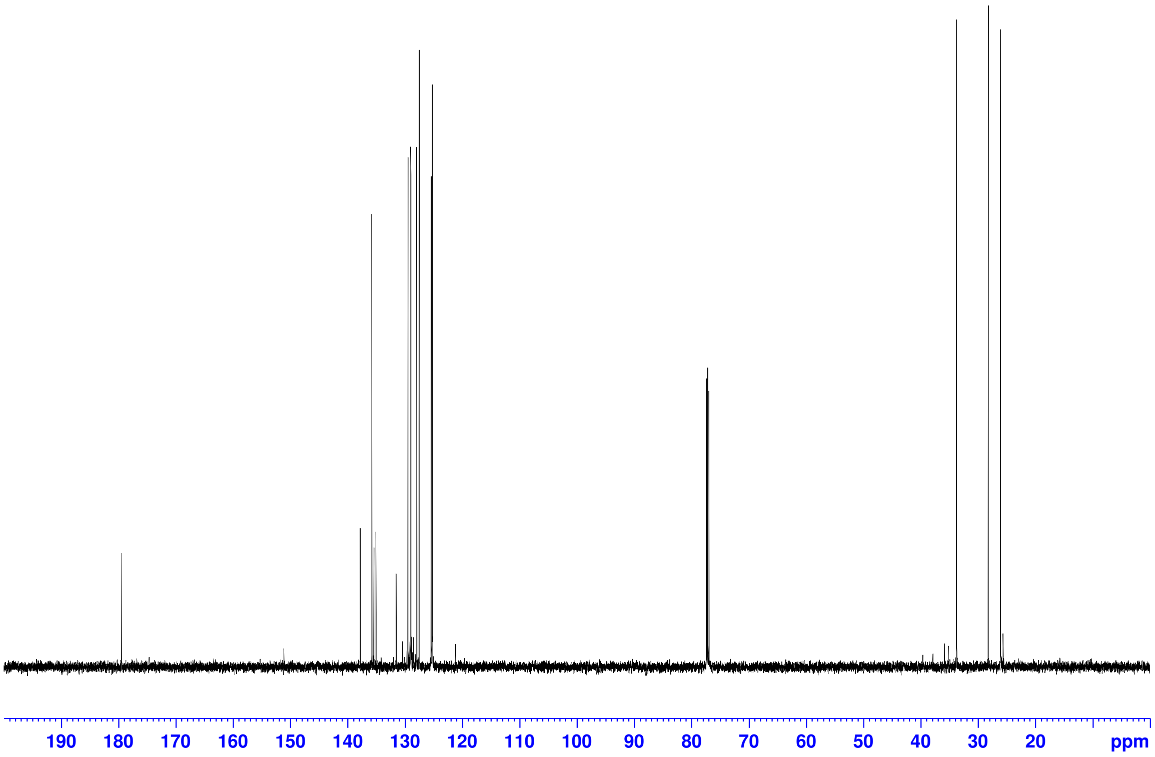

Supplementary Figure S14. ^{1}H NMR (500 MHz, CD_2Cl_2) and ^{13}C NMR (150 MHz, $CDCl_3$) of **3c.**

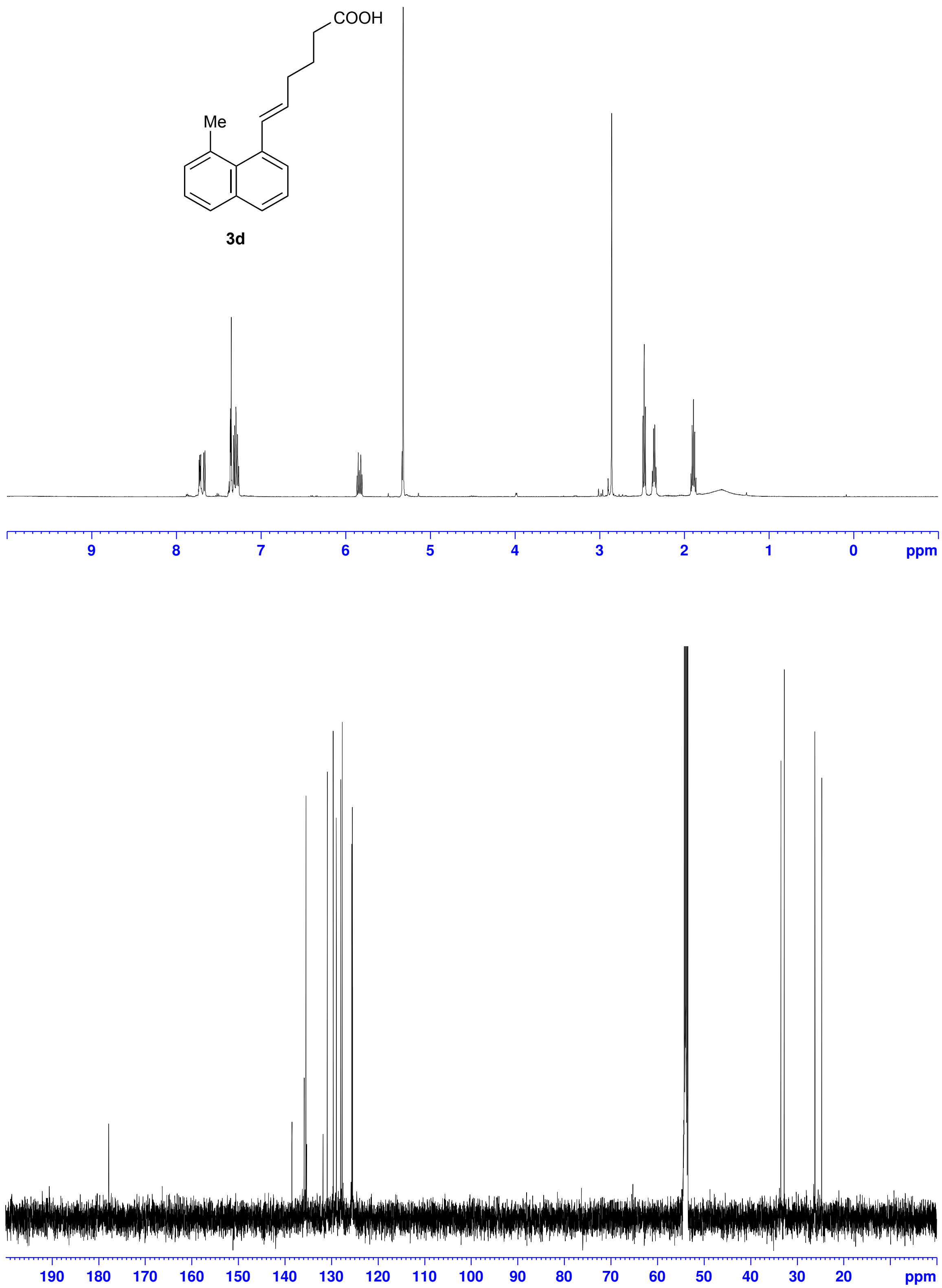

Supplementary Figure S15. ^{1}H NMR (500 MHz, CD_2Cl_2) and ^{13}C NMR (150 MHz, CD_2Cl_2) of **3d.**

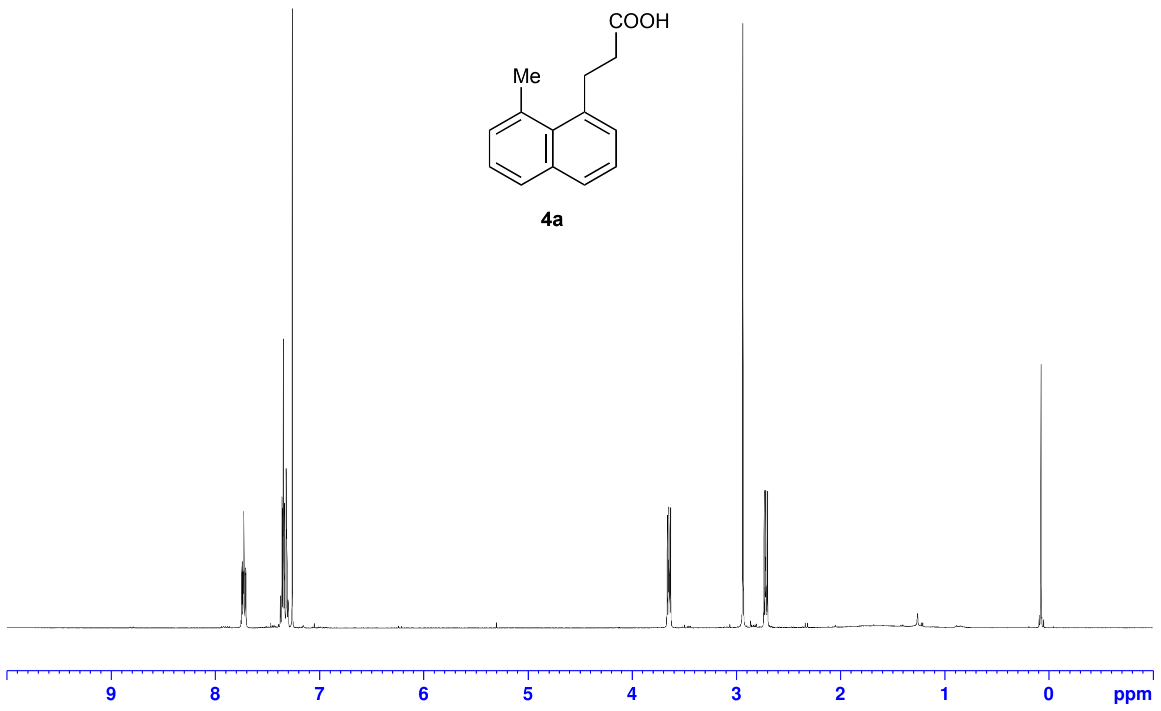

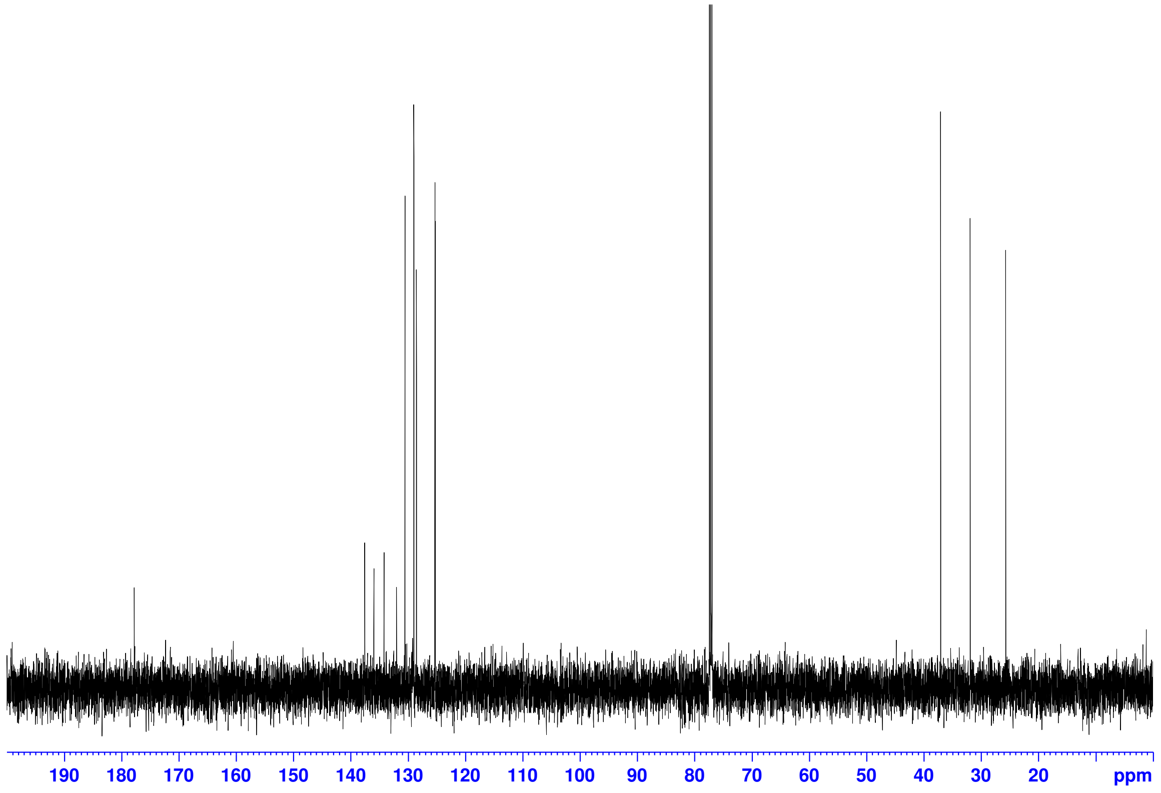

Supplementary Figure S16. ^{1}H NMR (500 MHz, $CDCl_3$) and ^{13}C NMR (150 MHz, $CDCl_3$) of **4a.**

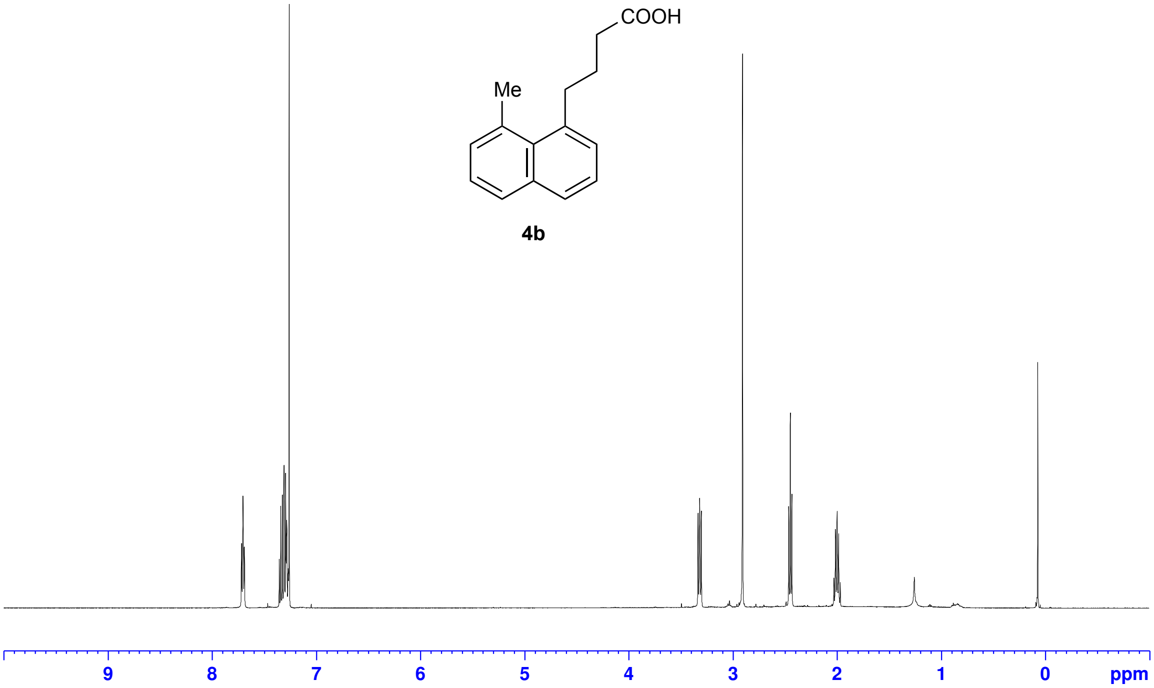

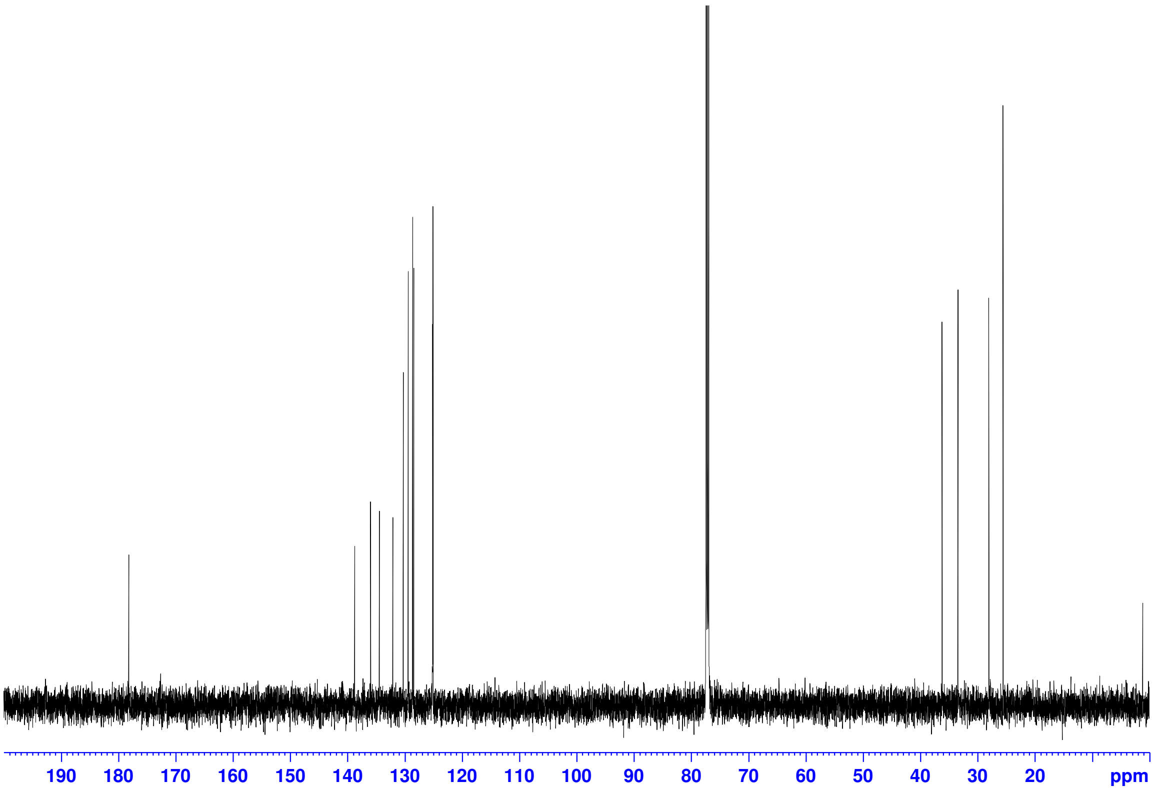

Supplementary Figure S17. ^{1}H NMR (500 MHz, $CDCl_3$) and ^{13}C NMR (150 MHz, $CDCl_3$) of **4b.**

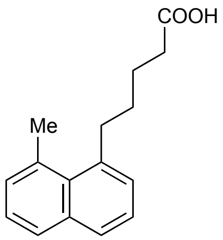

4c

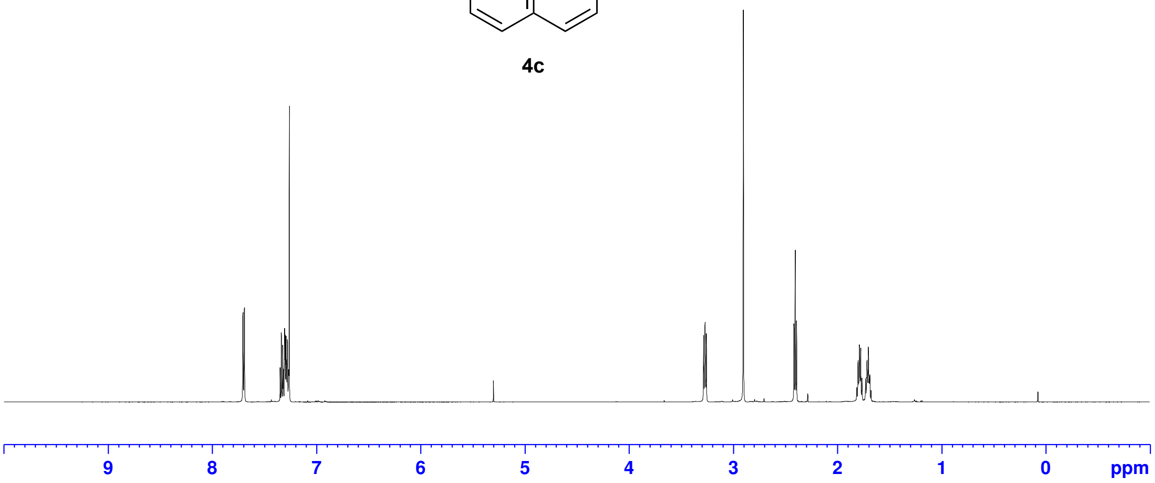

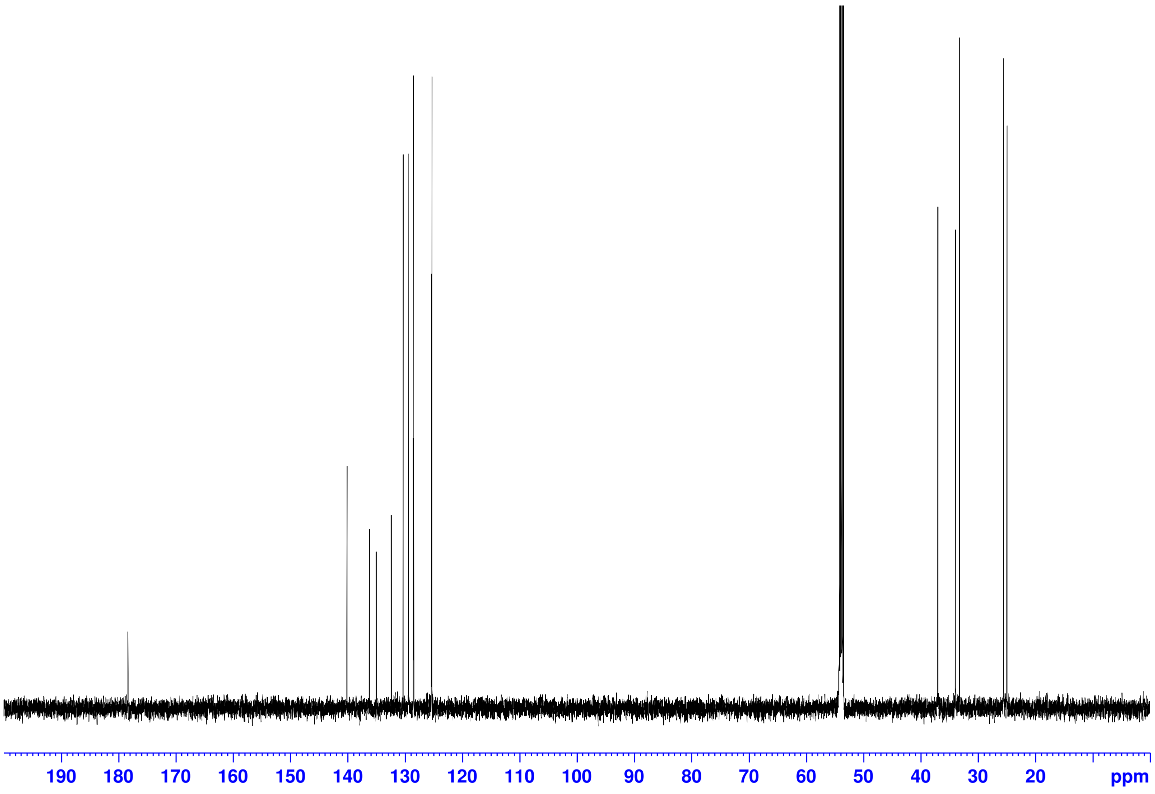

Supplementary Figure S18. ^{1}H NMR (600 MHz, $CDCl_3$) and ^{13}C NMR (150 MHz, CD_2Cl_2) of **4c.**

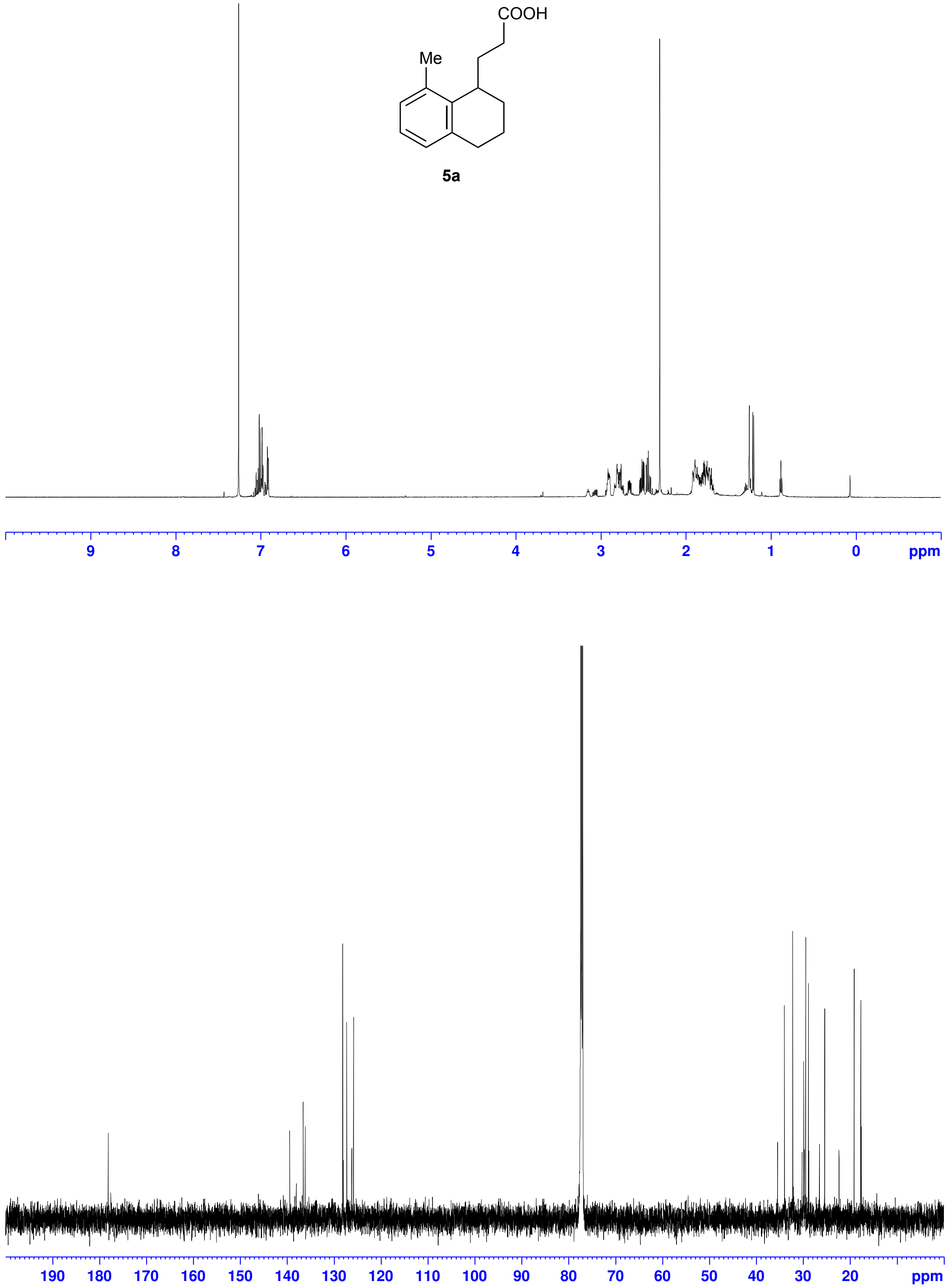

Supplementary Figure S19. ^{1}H NMR (600 MHz, $CDCl_3$) and ^{13}C NMR (150 MHz, $CDCl_3$) of **5a.**

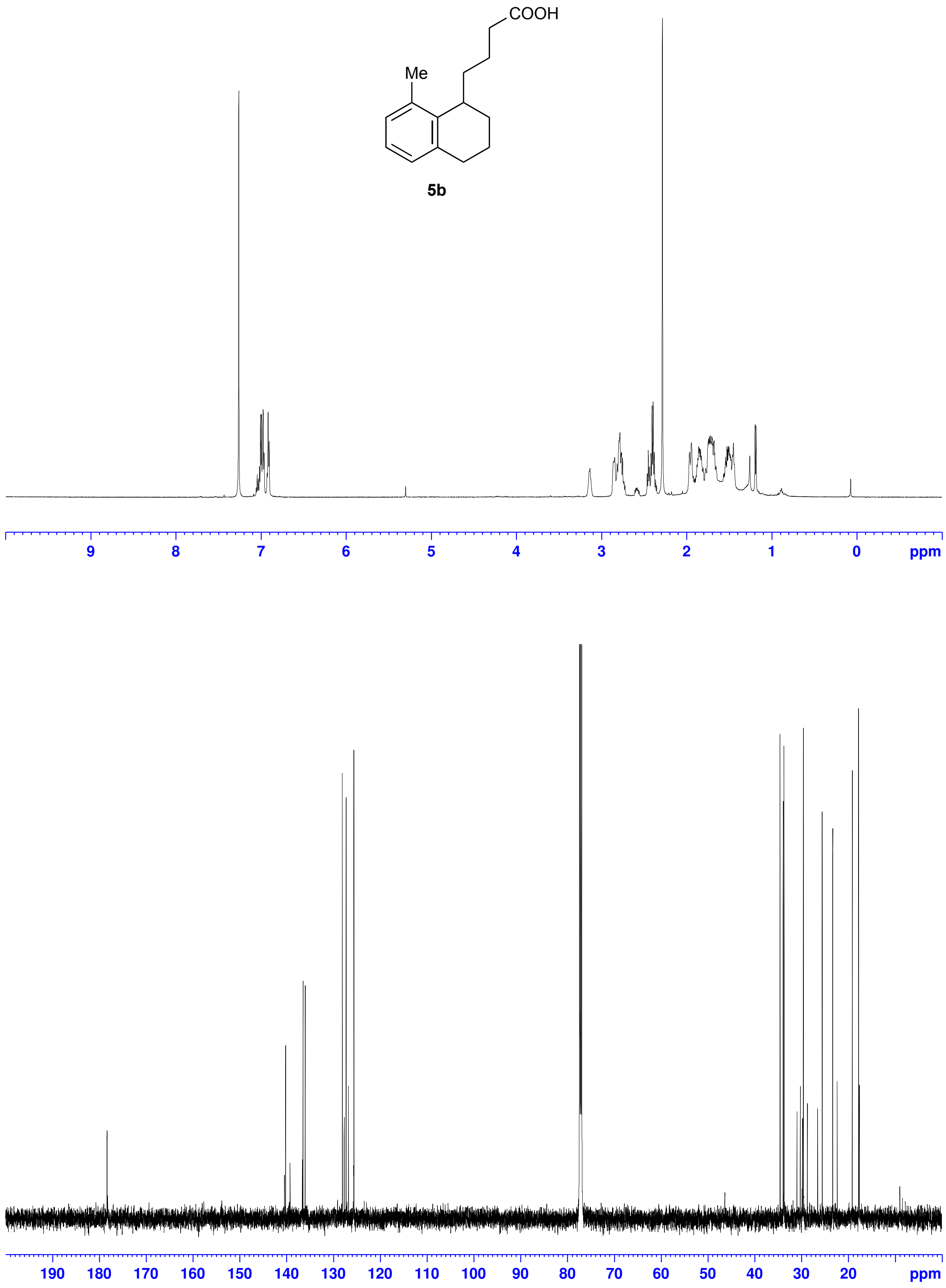

Supplementary Figure S20. ^{1}H NMR (600 MHz, $CDCl_3$) and ^{13}C NMR (150 MHz, $CDCl_3$) of **5b.**

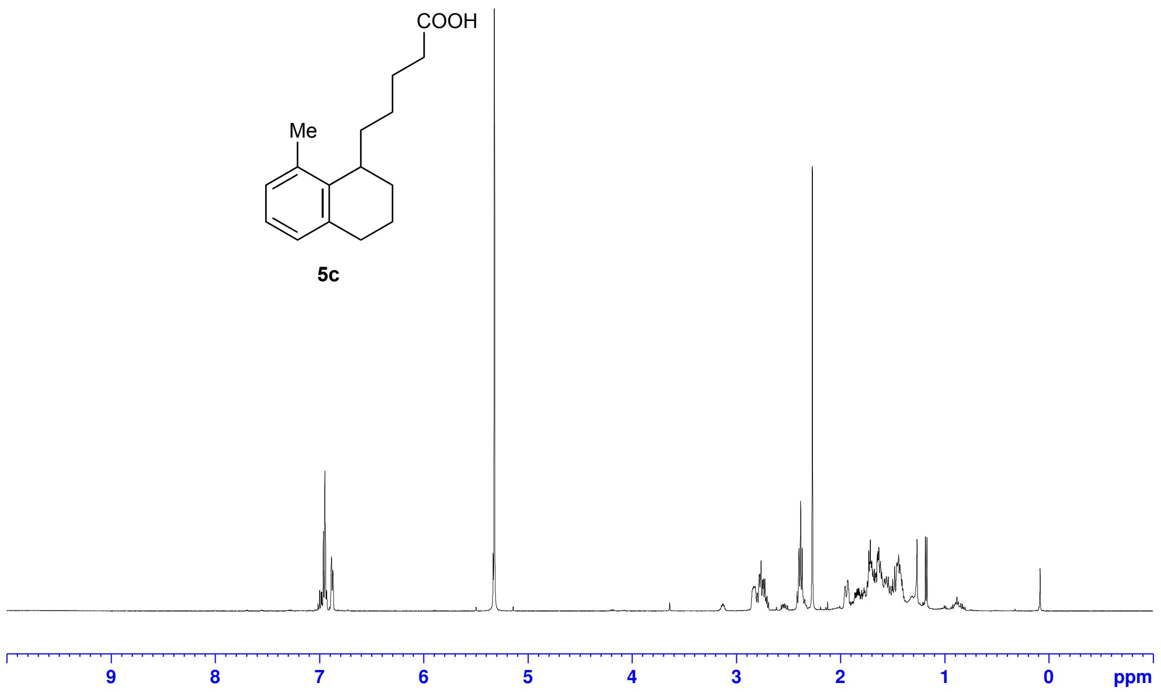

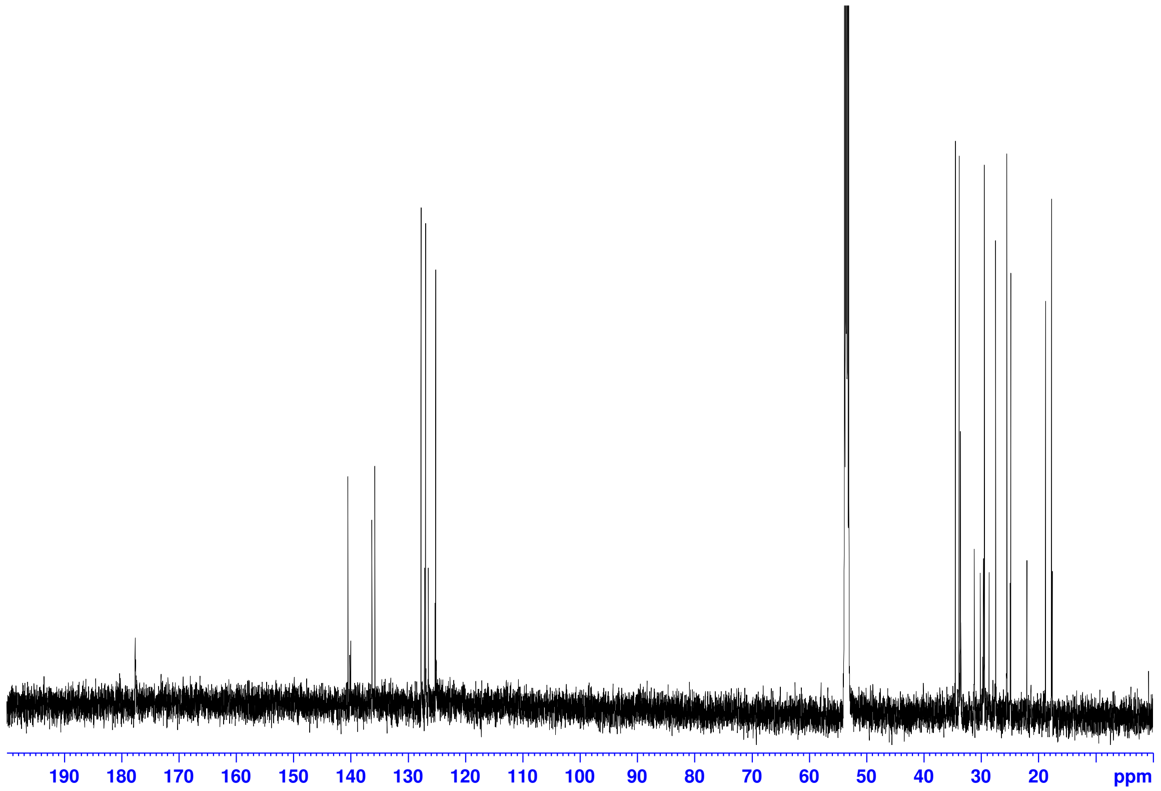

Supplementary Figure S21. ^{1}H NMR (500 MHz, CD_2Cl_2) and ^{13}C NMR (150 MHz, CD_2Cl_2) of **5c.**
